# Global Fe–O isotope correlation reveals magmatic origin of Kiruna-type apatite-iron-oxide ores

**DOI:** 10.1038/s41467-019-09244-4

**Published:** 2019-04-12

**Authors:** Valentin R. Troll, Franz A. Weis, Erik Jonsson, Ulf B. Andersson, Seyed Afshin Majidi, Karin Högdahl, Chris Harris, Marc-Alban Millet, Sakthi Saravanan Chinnasamy, Ellen Kooijman, Katarina P. Nilsson

**Affiliations:** 1 Section for Mineralogy, Petrology and Tectonics, Department of Earth Sciences, Uppsala University, Villavägen 16, 75236 Uppsala, Sweden; 2 Swedish Museum of Natural History, Dept. of Geosciences, Frescativägen 40, 114 18 Stockholm, Sweden; 3 Department of Mineral Resources, Geological Survey of Sweden, Villavägen 18, Box 670, 75128 Uppsala, Sweden; 4 Luossavaara-Kiirunavaara AB, Research & Development, FK9, 981 86 Kiruna, Sweden; 5 Geological Survey of Iran, Meraj St, Azadi Sq, Tehran, 138783-5841 Iran; 6 Geology and Mineralogy, Åbo Akademi University, Domkyrkotorget 1, 20500 Turku, Finland; 7 Department of Geological Sciences, University of Cape Town, Rondebosch, 7701 South Africa; 8 School of Earth and Ocean Sciences, Cardiff University, Park Place, Cardiff, CF10 3AT UK; 9 National Institute of Technology Rourkela, Department of Earth & Atmospheric Sciences, NIT Rourkela, Odisha, 769008 India; 10 Swedish Ministry of Enterprise and Innovation, Division for Business, Mäster Samuelsgatan 70, 10333 Stockholm, Sweden; 11 Present Address: Indian Institute of Technology (IIT) Bombay, Department of Earth Sciences, Powai, Mumbai, 400076 India

## Abstract

Kiruna-type apatite-iron-oxide ores are key iron sources for modern industry, yet their origin remains controversial. Diverse ore-forming processes have been discussed, comprising low-temperature hydrothermal processes versus a high-temperature origin from magma or magmatic fluids. We present an extensive set of new and combined iron and oxygen isotope data from magnetite of Kiruna-type ores from Sweden, Chile and Iran, and compare them with new global reference data from layered intrusions, active volcanic provinces, and established low-temperature and hydrothermal iron ores. We show that approximately 80% of the magnetite from the investigated Kiruna-type ores exhibit δ^56^Fe and δ^18^O ratios that overlap with the volcanic and plutonic reference materials (> 800 °C), whereas ~20%, mainly vein-hosted and disseminated magnetite, match the low-temperature reference samples (≤400 °C). Thus, Kiruna-type ores are dominantly magmatic in origin, but may contain late-stage hydrothermal magnetite populations that can locally overprint primary high-temperature magmatic signatures.

## Abstract (web summary)

The origin of giant Kiruna-type iron ores has been debated for nearly 100 years. This study employs extensive stable isotope data from Kiruna-type ores worldwide and magmatic and hydrothermal reference materials to show that iconic Kiruna-type ores originate primarily from ortho-magmatic processes.

## Introduction

**Fig. 1:**
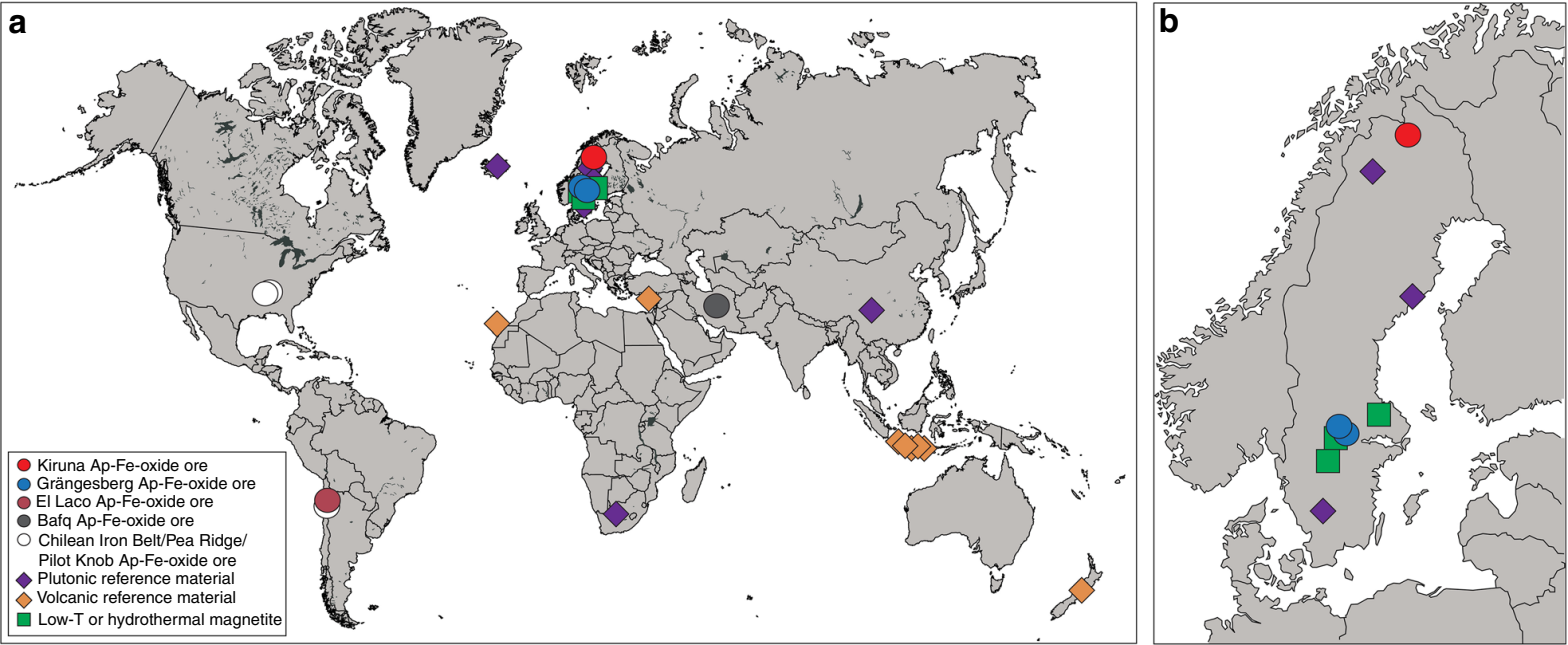
Sample overview map. **a** Global map showing the different locations of origin for apatite-iron-oxide ore and reference samples. **b** A close-up view of the main part of the Fennoscandian Shield showing the sample locations for magnetites from Sweden

Apatite-iron oxide ore is by far the biggest source of iron in Europe and one of the main iron sources worldwide^1^. In Europe, these magnetite-dominated ores have traditionally been sourced from two principal regions, the Bergslagen ore province in south central Sweden and the Kiruna-Malmberget region in northern Sweden (Fig. 1)^2^. The apatite-iron oxide ores from these localities are internationally renowned and similar ores elsewhere are usually referred to as being of Kiruna-type^3–5^. While the Grängesberg and Kiruna deposits are Palaeoproterozoic in age^6^, similar apatite-iron oxide deposits along the American Cordilleras are much younger and range in age from Jurassic to Neogene, like the Pliocene El Laco deposit in Chile^7–9^. Together with the occurrences of Paleozoic apatite-iron oxide ores in Turkey, Iran, and China, and Triassic examples from Korea^10–13^, apatite-iron oxide ores have repeatedly formed across the globe and throughout geological time.

The origin of the Kiruna-type apatite-iron-oxide ores remains ambiguous, however, despite a long history of study and a concurrently intense scientific debate. Several fundamentally different modes of formation have been proposed. Today two broad schools of thought prevail, represented by either direct magmatic formation processes, such as segregation or crystallization, or by hydrothermal replacement processes, including hydrothermal precipitation in the sense of iron-oxide-copper-gold (IOCG) deposits^3,4,14–31^. Specifically, the discussion revolves around a direct magmatic origin (ortho-magmatic) from volatile- and Fe–P-rich magmas or high-temperature magmatic fluids^1,5,18,31^, versus a purely hydrothermal one, where circulating, metal-rich fluids replace original host rock mineralogy with apatite-iron-oxide mineralizations at medium to low temperature^4,19,28–30,32^. An ortho-magmatic origin is generally understood to be either formation by direct crystallization from a magma or from high-temperature magmatic fluids (e.g. ≥800 °C), or via high-temperature liquid immiscibility and physical separation of an iron oxide-dominated melt from a silicate-dominated magma, where the former may subsequently crystallize as a separate body^17,18,33–38^. Hydrothermal processes, in turn, encompass transport and precipitation, including replacement-type reactions, by means of aqueous fluids at more moderate to low temperatures (typically ≤400 °C)^27,29,30,32^. Both the magmatic and the hydrothermal hypotheses are supported in part by field observations, textural relationships, and mineral chemistry, however, petrological field evidence and chemical trends of major and trace elements have frequently been interpreted in different ways^5,14,16–22,28–32^. Moreover, many of previous investigations have focused on one case study only and frequently present a range of various data, with individual data sets often being relatively restricted in respect to data volume. What has so far been missing is a broad and decisive geochemical approach to distinguish between these two rival formation hypotheses on an across-deposit scale. To date no systematic stable isotope study employing several distinct Kiruna–type apatite iron oxide ore deposits is available and, importantly, no systematic comparison with accepted magmatic and hydrothermal rock and ore suites has previously been presented in the literature.

## Results

### Scientific rationale and sample selection

Here we use the isotopes of iron and oxygen, the two essential elements in magnetite (Fe_3_O_4_), on magnetite samples from four major Kiruna-type ore provinces. Magnetite is the main iron-bearing component in Kiruna-type deposits and our approach therefore utilizes highly reliable major elements as petrogenetic tracers, as opposed to, e.g., traditional minor element approaches that rely on low-concentration constituents in these ores and their host rocks. We present an extensive set of new Fe and O isotope data of magnetite from a suite of world-class Kiruna-type ores from three continents, represented by Sweden, Chile, and Iran, and complement these data by a large suite of new comparative data on accepted magmatic and hydrothermal reference samples (54 new Fe–O coupled isotope ratios and another 12 individual ratios, totaling 120 new individual isotope ratios). In addition, the four different regions of Kiruna-type deposits investigated are separated in space and time, as are the extensive suite of volcanic, plutonic, and low-temperature reference materials that we employ to define the endmember processes reflected in our ore deposit data (see Fig. 1 and Supplementary Table 1). We use these data to address whether Kiruna-type apatite-iron oxide ores form primarily through direct magmatic processes (magma and magmatic fluids) at high temperatures (≥800 °C), or alternatively, through precipitation from hydrothermal fluids at considerably lower temperatures (≤400 °C). The comparative aspect of our work is a particular strength and to the best of our knowledge, no other systematic study with such global coverage has been performed to date. In addition, we offer the first substantial data sets for coupled Fe and O isotopes for the world famous Kiruna, Grängesberg, and Bafq deposits, plus new comparative data for El Laco that are consistent with published data for this deposit^5,14,17,18,32^. The study’s global extent, its systematic approach, its large data volume, and most crucially, the global Fe–O isotope correlation permits a decisive conclusion, which allows us to advance our understanding of the relationship between magmatic and hydrothermal processes in the genesis of Kiruna-type iron-oxide ore deposits. The use of a broad set of Fe–O isotope data is especially useful as it establishes not only genetic similarities between different deposits, but also provides constrains on the formation temperatures^1,5,39^ and allows us to identify a high-temperature versus a low-temperature origin of individual ore deposits.

**Fig. 2:**
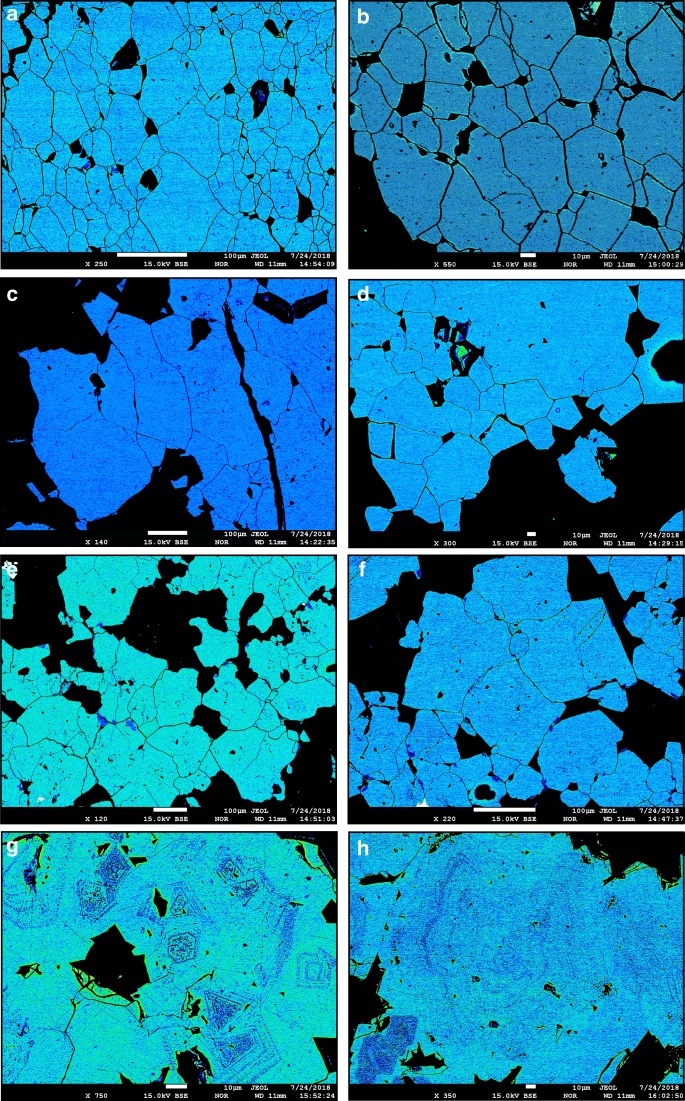
Images of apatite-iron oxide ores in this study. False-color BSE images of massive magnetite ore samples from **a**, **b** Kiruna; **c**, **d** Grängesberg; **e**, **f** Bafq; and **g**, **h** El Laco. Kiruna, Grängesberg, and Bafq magnetite samples are homogeneous and commonly lack zonation or signs of alteration. El Laco (**g**, **h**), is exceptional in this respect as for some samples intra-crystal zonation is observed. As a supplement, regular greyscale BSE images for these samples are provided in Supplementary Fig. 1

To facilitate a widely applicable comparison of apatite-iron-oxide ores, analyses of iron and oxygen isotope ratios of magnetite from massive apatite-iron-oxide ores from the Kiruna Mining District in northern Sweden (*n* = 14), the Grängesberg Mining District and the nearby Blötberget ore body in central Sweden (*n* = 16), the El Laco district in Chile (*n* = 6), and the Bafq Mining District in central Iran (*n* = 6; Figs. 1 and 2) were performed. These ore provinces and regions are briefly introduced below, and full details are given in Supplementary Note 1.

The study of apatite-iron-oxide mineralizations began at the iconic deposits of the Kiruna Mining District^3^. The present Kiruna mine at Kiirunavaara is the largest deposit of its type, and is the main supplier of iron ore in Europe. This deposit alone represents a pre-mining reserve of ≥2 billion tons of high grade ore. The ores in Kiruna and similar Palaeoproterozoic deposits in the district (Supplementary Table 1) are dominated by magnetite and contain between 50 and 70% Fe with a P content of, commonly, up to 2 wt.% that is mainly hosted by fluorapatite and subordinate monazite-(Ce)^25^. Additional gangue minerals that can occur in small proportions are Mg-rich actinolite, phlogopite, chlorite, titanite, talc, feldspar, quartz, carbonates, sulfides, sulfates, and clays^3,5,25^.

The apatite-iron-oxide ores of the Grängesberg and the nearby Blötberget deposits represent the largest iron-ore accumulation in the Bergslagen ore province, a classic mining region in central Sweden considered to represent a Palaeoproterozoic continental rift or back arc basin^40^. A historic production of 156 Mt of ore, averaging 60% Fe and 0.81% P, is documented from Grängesberg. In addition to iron oxides (dominantly magnetite), the presence of phosphates such as fluorapatite, monazite-(Ce), and xenotime-(Y), together with REE-silicates constitute a potentially significant P and REE resource^1,41^.

The El Laco apatite-iron-oxide ore deposit at the Pico Laco volcanic complex in northern Chile consists of seven individual ore bodies which together comprise ~500 Mt of mainly magnetite-dominated ore with an average grade of 60% Fe^5,18,26,30^. The Plio- to Pleistocene El Laco deposit is part of the young apatite-iron-oxide mineralizations that characterize the eastern high Andes and is separate from the Cretaceous apatite-iron-oxide ores of the so-called Chilean Iron Belt^7,14,17,18^.

The Bafq Mining District in Central Iran comprises 34 documented iron ore mineralizations with a total reserve of ~2 billion tons of iron ore with grades between 53 and 65%^11,42^. The apatite-iron oxide ores of the Bafq region are coeval with their early Cambrian andesitic and rhyolitic host rocks that formed in a volcanic arc setting^43^. Samples from the Bafq Mining District were collected from the Sechahun, Lakkeh Siah, Chadormalu, and Esfordi deposits^42^.

Analyzed plutonic reference samples (Fig. 1) include magnetite from the layered igneous intrusion of Panzhihua in China (*n* = 2), the Bushveld igneous complex in South Africa (*n* = 1), and the layered intrusions of Taberg (*n* = 1), Ulvön (*n* = 1), and Ruoutevare (*n* = 1) in Sweden and an iron-rich gabbro nodule from Iceland (*n* = 1). Samples representative of magmatic magnetites of volcanic derivation were chosen from basalts and dolerite from the Canary Islands (*n* = 3), recent basaltic andesites from Indonesia (*n* = 6), dacites from New Zealand (*n* = 2) and a hypabyssal dolerite from Cyprus (*n* = 1; Supplementary Table 1). Reference samples for low-temperature or hydrothermal iron ore deposits include magnetite from the polymetallic magnetite-skarn deposit at Dannemora (*n* = 4), the banded iron formation at Striberg (*n* = 1), and the marble-hosted iron oxide deposit at Björnberget (*n* = 1), all situated in Bergslagen, Central Sweden (Supplementary Table 1, Supplementary Note 1).

Our interpretations are based on the combination of new iron (*n* = 63) and oxygen (*n* = 57) isotope ratios combined with literature data for magnetite from apatite-iron-oxide ores and available volcanic, plutonic and the low-temperature or hydrothermal reference materials^1,5,14,31,32,44–48^. Notably, the literature data for low-temperature or hydrothermal magnetite include a sample from the north-American Mineville apatite-iron-oxide deposit, which has been extensively overprinted by later hydrothermal processes^14,49^.

### Iron and oxygen isotope results

**Fig. 3:**
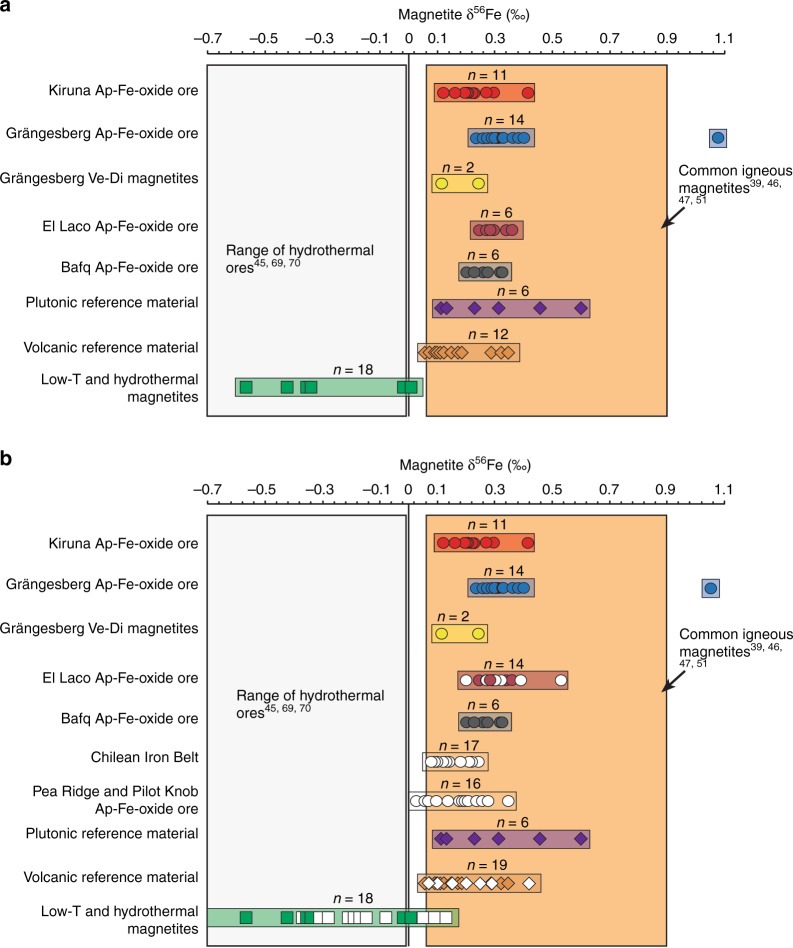
Iron isotope results. Shown is **a** the distribution of iron isotopes in magnetites from the Kiruna and Grängesberg districts, El Laco, and the Bafq district from this study, and **b** our data together with available literature data^14,45,47,48^. Reference fields for common hydrothermal and magmatic magnetites are shown for comparison^39,45–47,51,69,70^. Magnetites from apatite-iron oxide ores show a clear distinction from low-temperature or hydrothermal magnetites and overlap with the layered intrusions and volcanic reference magnetites (i.e. in the magmatic reference field). Data from Wang et al.^45^ show the effects of a progressive transgression from ortho-magmatic processes to hydrothermal fluid evolution from originally higher to lower δ^56^Fe values and an originally magmatic fluid may thus evolve into a hydrothermal fluid. One hydrothermal sample from the highly altered, remobilized, and recrystallized Mineville deposit in the USA (δ^56^Fe = −0.92‰, δ^18^O = −0.79)^14^ is not shown for simplification. Ve-Di samples represent vein and disseminated magnetites

Magnetite from massive apatite-iron-oxide ores from Kiruna have a relatively restricted δ^56^Fe range of +0.12 to +0.41‰ (*n* = 11) (Supplementary Table 1, Fig. 3). The Grängesberg and Blötberget magnetite samples show δ^56^Fe-values mainly between +0.11 and +0.40‰ (*n* = 16). However, one sample from Grängesberg shows an exceptionally high value of +1.0‰. Apatite-iron-oxide ores from El Laco yield δ^56^Fe-values between +0.24 and +0.36‰ (*n* = 6). Magnetite from Bafq have a range in the +0.20 to +0.32‰ interval (*n* = 6).

Magnetite samples from the plutonic and volcanic reference suites show δ^56^Fe-values from +0.11 to +0.61‰ (*n* = 5) and from +0.06 to +0.46‰ (*n* = 13), respectively, consistent with iron isotope values for magmatic rock suites elsewhere (e.g. Figure 3)^39,46^.

The low-temperature deposit group, i.e., the Dannemora iron oxide skarn samples and the Björnberget and the Striberg samples, on the other side, show relatively low δ^56^Fe-values that range from −0.57 to +0.01‰. The magnetite compositions in the low-temperature group form a separate group (Fig. 3) that does not overlap with the reported range of igneous magnetites (+0.06 to +0.49‰)^39,46^, but with low-temperature hydrothermal samples from elsewhere (e.g., Mineville, USA and Xinqiao, China)^14,45,49^.

**Fig. 4:**
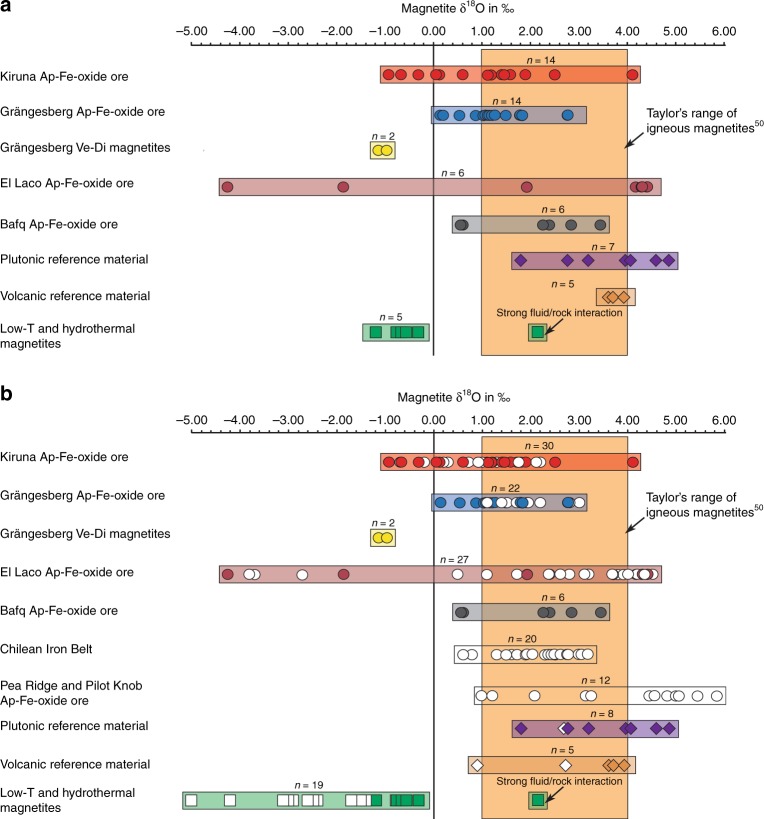
Oxygen isotope results. **a** Oxygen isotopes of magnetite samples from Kiruna, Grängesberg, El Laco, and the Bafq district from this study are compared to reference samples from layered igneous intrusions, recent volcanic magnetites, and low-temperature or hydrothermal ore deposits as well as **b** data from the Chilean Iron Belt^14^, the Pea Ridge and Pilot Knob deposits^48^. The range of typical igneous magnetite is outlined in the reference box^50^. The majority of magnetite samples from apatite-iron oxide ores plot within the reference field for common magmatic δ^18^O-values and overlap with magnetite values from recent volcanic rocks and layered intrusions. The low-temperature or hydrothermal reference suite, together with low-temperature magnetite literature data^14,44^, plot dominantly to the left of the magmatic magnetite field, with only one exception, a magnetite from the Fe-skarn deposit at Dannemora. This particular outlier comes from a part of the deposit (Konstäng) which itself represents a geochemical anomaly within the Dannemora deposit. Our values for El Laco overlap with results from previous studies^5,14,17,18,32^

Magnetite separates from the Kiruna Mining District range in δ^18^O value from −1.0‰ to +4.1‰ (*n* = 14), and those from Grängesberg and Blötberget are between −1.1 and +2.8‰ (*n* = 16). (Supplementary Table 1, Fig. 4). Magnetite from El Laco shows a large range in δ^18^O values from −4.3 to +4.4‰ (*n* = 6), whereas magnetite samples from Bafq give a smaller range of +0.6 to +3.4‰ (*n* = 6).

Magnetites from the plutonic reference samples (Panzhihua, Bushveld, Taberg, Ulvön, Ruoutevare, and Iceland gabbro bomb; *n* = 7), and from recent volcanic provinces (New Zealand, Indonesia and Tenerife, *n* = 3) show exclusively positive δ^18^O-values between +1.8 and +4.8‰, and +3.7 to +3.9‰ respectively, which is within or near the commonly accepted range of igneous magnetites (δ^18^O = +1.0 to +4.0‰)^50^. The low-temperature and hydrothermal reference ore samples (e.g. Dannemora, Björnberget, and Striberg), have low δ^18^O values (−1.2 to −0.4‰; *n* = 5) with one exception; a skarn sample from Dannemora that has a δ^18^O-value of +2.1‰ (Supplementary Table 1). This particular sample, however, comes from a part of the deposit (Konstäng) which itself is reported to be geochemically anomalous with respect to the deposit as a whole (see Supplementary Note 1).

Comparing the oxygen and iron isotope data of magnetite samples from Kiruna, Grängesberg, El Laco, and Bafq, we find that they overlap with the magnetite data from the plutonic and recent volcanic reference samples. Recognized low-temperature or hydrothermal deposits, such as Striberg, Björnberget, and Dannemora record magnetite isotope values that, in turn, differ distinctly in their Fe and O isotope signatures from magmatic values (Figs. 3 and 4). We note that the oxygen isotope data from two vein and disseminated (Ve-Di) magnetite samples from Grängesberg, three samples from Kiruna, as well as magnetite from two samples from El Laco overlap with the low-temperature and hydrothermal reference group. However, these samples still show Fe isotope signatures that are similar to our magmatic reference suite and are hence assumed to reflect originally igneous sources.

**Fig. 5:**
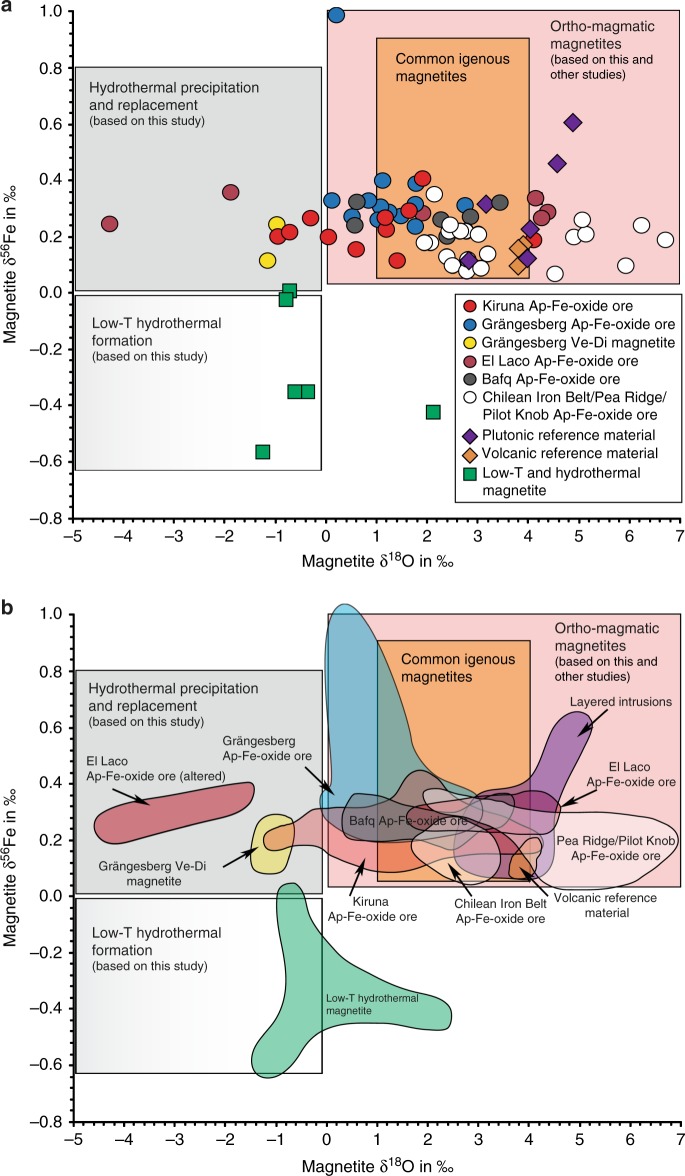
Distribution of Fe and O isotope values of magnetite samples used in this study. **a** The various magnetite samples can be divided into three groups according to their Fe–O isotope composition; (i) high-temperature magmatic magnetites, (ii) hydrothermal magnetite samples, and (iii) low-temperature magnetite samples. **b** Most of the magnetite compositions of the apatite-iron-oxide ores in this study lie within, or near, the reference field for igneous magnetite, and overlap with the plutonic and volcanic magnetite samples analysed as reference suite. See also Supplementary Note 1 and Supplementary Fig. 2 for a detailed assessment of temperature-dependent equilibrium compositions. Reference field for common igneous and hydrothermal magnetites are based on literature data^14,39,44,46,47,50,51^

The compositional overlap between Kiruna-type magnetite and the plutonic and volcanic reference suite for Fe and O isotopes is consistent with an ortho-magmatic (magma or highest-temperature magmatic-fluids) origin for the Kiruna-type apatite-iron-oxide samples in this study. Low-temperature processes are reflected in a small number of the Kiruna-type ore samples (*n* = 7) represented by vein- and disseminated-type magnetite samples. The exceptionally high Fe-value for one Grängesberg sample, in turn, is in agreement with the “ultra-magmatic” Fe isotope composition recorded in magnetite from the Bushveld complex (Fig. 3)^51^. In contrast, the lower δ^56^Fe- and δ^18^O-values in Figs. 3 and 4 then either reflect the lower end of the magmatic temperature range, or secondary effects, such as alteration, as well as leaching and subsequent re-precipitation at temperatures below 400 °C, which postdates an initial high-temperature (magmatic) stage of formation (Fig. 5). Therefore, the oxygen and iron isotope data for the massive apatite-iron oxide magnetites indicate an originally high-temperature magmatic signature that, most clearly for oxygen isotopes, transitions to lower temperature values indicating a gradual cooling trend. One critical issue, especially for the Palaeoproterozoic Swedish deposits, which have gone through variable grades of metamorphism, is that post-depositional processes (e.g., fluid overprint, re-heating) might have affected the primary isotope composition of the ore and cannot be entirely excluded. Yet, since the same trends in isotope signatures are observed for both the older and younger, less geologically overprinted deposits, we argue that post-depositional changes in isotope composition was negligible in respect to the Fe–O isotope chemistry of our magnetite samples. This is particularly the case for the massive magnetite ores, where this overall chemically inert and refractory mineral would provide local buffering with regards to post-depositional re-equilibration^1^.

## Discussion

Based on fluid and melt inclusion studies and isotope compositions of mineral pairs, high temperatures of ore formation have been proposed for various apatite-iron oxide ores. For instance, temperature determinations for equilibrium magnetite–quartz pairs and magnetite–pyroxene pairs from Kiruna, El Laco, and Grängesberg yield temperatures that consistently exceed 600 °C^1,5^. Such crystallization temperatures are further supported by, e.g., the occurrence of high-temperature actinolite in, e.g., the Los Colorados and Kiruna deposits^14,34,52^ by Ti exsolution textures in magnetite from Kiruna^52^, and by Zr in titanite studies at Kiruna that suggest 750–800 °C^53^. These temperature determinations provide independent support for a high-temperature (ortho-magmatic) origin of these ore assemblages. Using these temperatures as a reference point and employing appropriate equilibrium fractionation factors, we modeled the isotope compositions of respective equilibrium sources to test the mineralization conditions of our sample suite (Supplementary Table 2 and Supplementary Fig. 2). We applied available fractionation factors between magnetite and andesite/dacite magma (T~1000 °C) as well as between an equilibrium aqueous magmatic fluid phase at temperatures between 600 and 800 °C for both iron and oxygen isotopes (see Supplementary Tables 2, 3, 4, 5).

For oxygen isotopes, the calculations indicate that magnetite samples from apatite-iron oxide ores with δ^18^O ≥ 0, corresponding to over 80% of our sample set, reflect equilibrium with either an intermediate magma (δ^18^O of +5.7 to +8.7‰) or a high-temperature magmatic fluid (δ^18^O of +5.2 to +9.6‰; Supplementary Tables 2, 3, 4, and 5, Supplementary Fig. 2). Although equilibrium with ortho-magmatic, high-temperature sources are found for most of our samples, it naturally remains difficult to distinguish between a magma or a very high-temperature, magmatically derived aqueous fluid as the initial magnetite source. This realization is highlighted by the fact that our oxygen isotope values from Kiruna-type magnetite samples overlap with oxygen isotopes from the Granisle porphyry copper deposit presented in Bilenker et al.^14^, which is proposed to have formed entirely from expelled ortho-magmatic fluids. For the same samples, iron isotope equilibrium source calculations yield values that correspond to magmas and modeled magmatic fluids with δ^56^Fe values from +0.08 to +0.38‰, and −0.13 to +0.17‰, respectively, which plot partly above the reported array of intermediate magmas and magmatic waters (Supplementary Fig. 2)^39,54^. This suggests that the metal sources of the samples that exceed the range were likely enriched in the heavy iron isotope (^56^Fe) already at the time of magnetite formation^39,46^, which we refer to as ultra-magmatic. Such ^56^Fe enrichment may be caused by magmatic degassing, which is common in many volcanic systems^39^, or more likely represents the result of iron oxide-enriched melts acting as an iron sink^55^. Notably, this enrichment is also seen in some samples of our magmatic (plutonic and volcanic) reference suite (Supplementary Table 4, Supplementary Fig. 2), as well as in several Kiruna-type apatite-iron-oxide derived magnetite samples in other studies (Supplementary Table 5, Supplementary Fig. 2)^14,48^. Magma degassing may preferentially remove the lighter Fe isotopes^39^, increasing the δ^56^Fe in the melt. Alternatively, the iron-sink scenario is possibly caused by the tendency for magnetite to incorporate the heavy Fe isotopes over the lighter ones either during crystallization or during silicate-metal immiscibility. This could lead to ultra-magmatic signals following prolonged crystallization of silicate phases from a basaltic magma. Specifically, the heavier iron isotope that partitions into the melt due to removal of early Fe-fractionating minerals, such as olivine and pyroxene, will deplete the melt in the isotopically lighter Fe^2+^ and will leave Fe^3+^ preferentially in the residual magma^46^. When magnetite becomes the dominant iron-bearing phase later in the crystallization sequence it will consequently reflect the isotopically heavy melt signature^46^. Finally, the high Fe^3+^/Fe_tot_ and the strong bonding in the tetrahedral site for Fe^3+^ in magnetite make it a highly suitable host for the heavier iron isotope^46^. The combined effects of magma degassing, prolonged fractional crystallization leading to more andesitic to dacitic melts, and the preference of magnetite for the heavy iron isotope are the likely reasons for the ultra-magmatic signature in several magnetite samples from the plutonic-volcanic reference suite as well as from some apatite-iron-oxide ore samples.

The key concepts proposed by the magmatic school of thought are formation of Kiruna-type ores by either liquid immiscibility or separation of magnetite cumulates (by sinking or flotation/frothing) from a silicate melt^5,17,18,31,38,48^. To evaluate which magmatic process is dominantly responsible for the formation of the massive magnetite bodies has proven difficult and while some petrological and experimental studies favor the concept of liquid immiscibility^18,38^, other workers suggest cumulate-type processes^14,31^. Unfortunately, the currently available experiments that support liquid immiscibility are not truly representative of nature (e.g. 40 wt.% P_2_O_5_ in a starting melt)^36,38^. Such a composition contrasts with the host rocks to actual Kiruna-type deposits at Bafq, El Laco, Grängesberg, and Kiruna. Using our new data to assess formation mechanisms, we can employ the isotope fractionation between immiscible Fe-rich melts and their silicate counterparts^35,56^. Assuming a hydrous system, the maximum fractionation for oxygen isotopes between an iron-rich melt and a silicate melt is 0.8‰^35^. Testing if our massive magnetite samples are representative of an Fe-rich melt that formed from immiscibility would require associated equilibrium silicate melts with δ^18^O between −3.5 and +5.2‰. These values are out of the range of common intermediate igneous rocks^57,58^. Testing the same approach for the plutonic reference material (*n* = 7) produces two equilibrium melts (δ^18^O = +5.4 and +5.6‰) that both match a basaltic igneous composition (MORB = +5.7 ± 0.4‰). The formation of Kiruna-type ores by liquid immiscibility is therefore not fully aligned with our data. Although this would, at first glance, favor cumulate processes over liquid immiscibility, there is probably uncertainty as to the natural fractionation of δ^18^O during liquid immiscibility and, to date, little information is available for Fe–isotopes in such situations. This leads us to encourage further tests in order to verify which of these two magmatic processes is more dominant in the formation of apatite-iron oxide systems. Future developments in the field of in-situ analysis of Fe–O isotope composition may hopefully help resolve small features such as thin, Ti-poor outer alteration rims as reported by e.g., Knipping et al.^31^ on magnetite samples from the Los Colorados apatite-iron-oxide deposit that these authors interpret to have bearing on the ore formation process. However, such textures cannot yet be analyzed for Fe and O isotopes in situ^59^, and in respect to our whole-grain results, such volumetrically small features would have a minimal effect on the bulk isotope signature of our samples.

In contrast to the massive ore samples, the vein and disseminated magnetites associated with apatite-iron oxide ores and low-δ^18^O massive magnetite ores from Kiruna, Grängesberg, and El Laco (i.e. δ^18^O_mgt _< 0‰, *n* = 7) are not in equilibrium with recognized magmatic sources at the previously established temperatures. For these samples equilibrium with magmatic sources would only be obtained at temperatures below 400 °C (Supplementary Table 6). Notably, Fe–P-rich magmas can only be liquid down to ~600 °C^34^ implying that these magnetite samples cannot have formed directly from a magma. Moreover, the O isotope signatures in these magnetites overlap with those of our low-temperature and hydrothermal reference group and they can either be explained by a cooling magmatic fluid, or by a low-temperature hydrothermal system with external fluid influx. For El Laco, disequilibrium between high-temperature magmatic sources and a sub-set of low-δ^18^O samples has previously been discussed and is attributed to late-stage or secondary processes^5,32^. The low-δ^18^O magnetite samples from El Laco are also associated with considerable amounts of hematite that probably formed as a result of oxidation of magnetite by low-δ^18^O, possibly meteoric-dominated, hydrothermal fluids at temperatures of ≤150 °C^5,32^. Meteoric fluids, particularly from higher altitudes such as the Andes, could cause such a negative shift in oxygen isotopes. However, these fluids would be Fe poor and may thus have only limited effect on iron isotope composition. For the isotope analysis great care was taken to avoid any direct hematite contamination of the samples, yet minor hematite formation along fractures in discrete magnetite grains is seen in some El Laco samples and may in part explain the larger spread in oxygen isotope data^5,14,17,18,32^. Our iron isotope data, on the other hand, confirm a magmatic isotope signal throughout, i.e., even in the low-δ^18^O samples. The low-δ^18^O but magmatic δ^56^Fe magnetite compositions at El Laco are thus likely to represent overprint, remobilization, and reprecipitation of an originally magmatic iron and oxygen signal by hydrothermal fluids that strongly affected oxygen isotopes in the magnetite, but had little effect on the iron isotopes^14,31^. The fluids affecting the ores may either have been derived from the cooling magmatic system or from an external fluid contribution during the evolution of the mineralization. Remarkably, hydrothermal overprint in the Kiruna Mining District in Sweden, for instance, has now been suggested to post-date ore formation with up to 250 My^52^. If this is correct, it implies that some non-magmatic (i.e. hydrothermal) isotope signals in apatite-iron-oxide ores may be entirely unrelated to the original mode of formation.

**Fig. 6:**
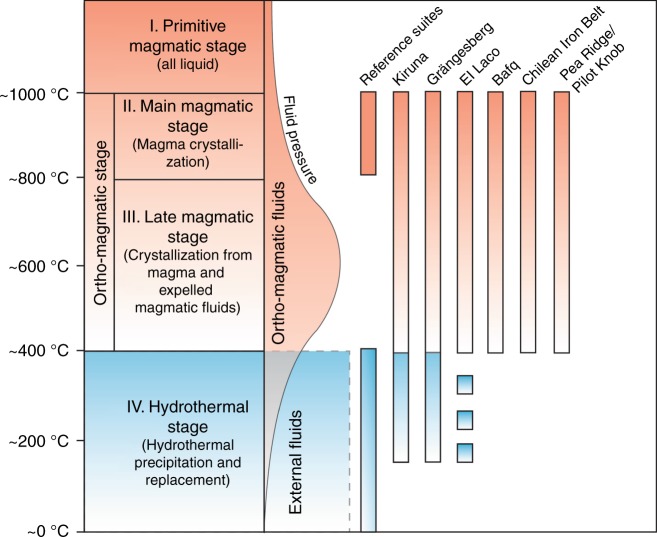
Schematic representation of magmatic stages for Kiruna-type apatite-iron-oxide ores from this and other studies, and from the analyzed reference materials. Stages II and III comprise ortho-magmatic ore formation: with decreasing temperature and on-going crystallization in the melt, the volatile/fluid pressure will increase and magmatic fluids are being expelled into the surrounding rocks. Below ~600 °C (towards the end of stage III), the magmatic-derived volatile pressure may begin to decrease, allowing progressively more of available external fluids into the system that initiate hydrothermal activity (<400 °C). Massive apatite-iron oxide ores appear to commence crystallization in the ortho-magmatic stages (Stages II and III), whereas vein and disseminated magnetites formed mainly during Stage IV (hydrothermal precipitation and replacement). This implies that the commonly observed hydrothermal signals in apatite-iron oxide ores are late-stage products that are results of syn- to post-magmatic hydrothermal processes active during the cooling of the volcanic system, or in some cases possibly reprecipitation during later overprints

The Fe–O isotope data obtained on magnetite samples from apatite-iron oxide ores from Sweden, Chile, and Iran are thus broadly consistent with the few available Fe–O isotope values from (a) the literature (apatite-iron-oxide ores from USA and the Chilean iron belt), and (b) the volcanic and plutonic reference data in this study from various layered igneous intrusions (from China, South Africa and Sweden) and from recent volcanic provinces (Indonesia, Canary Islands, New Zealand, and Iceland). Moreover, the iron and oxygen isotopes of magnetite samples from apatite-iron oxide ores differ, for most samples, from magnetites produced by low temperature or hydrothermal processes (≤400 °C). The iron and oxygen isotope data together with the calculated equilibrium sources are therefore in agreement with a predominantly (ortho-)magmatic origin (magma and high temperature magmatic fluids) for the investigated Kiruna-type deposits, rather than of a low-temperature hydrothermal one. While our data are very well suited to distinguish these broad formation conditions, they are not ideally suited to resolve the precise formation process and agent, i.e, we cannot distinguish magma versus high-temperature fluid or liquid immiscibility processes versus magnetite accumulation (Supplementary Fig. 2). Accepting an essentially magmatic nature of these deposits, a local hydrothermal overprint and replacement within a volcanic to sub-volcanic system would naturally be expected (Fig. 6), and will involve localized late-stage or secondary hydrothermal alteration and overprint^1,18,32,41^. Hydrothermal alteration might locally be pronounced and may seem pervasive in places, as is known from many volcanic provinces^60,61^. This by-product of otherwise ortho-magmatic formation processes must, however, not be confused with the main source signal of most Kiruna-type magnetite samples revealed in our study (Figs. 5 and 6).

Hydrothermally formed magnetite is isotopically distinct and appears subordinate in our sample suite. The bulk of the Kiruna-type ore investigated thus formed in sub-volcanic environments under essentially high-temperature magmatic conditions, in line with the magmatic school of thought^3,5,14,17,18,31,62^. Precipitation of magnetite was either from iron-oxide-saturated intermediate magmas or from immiscible Fe-rich melts that separated from broadly andesitic to dacitic parent magmas and subsequent physical (either gravity or gas-driven) magnetite segregation to form massive magnetite melts and mushes^18,24,31,35,63^. Kiruna-type apatite-iron-oxide ores are hence dominantly a magmatic phenomenon and they presumably continue to form in active arc- and back-arc type sub-volcanic environments up to the present day. Our combined isotope data and calculations represent a significant advance in the understanding of Kiruna-type ore deposits and over-rules most arguments for a completely hydrothermal mode of formation. Moreover, we provide a reference system for Fe–O isotopes in Kiruna-type ores against which future research can test genetic concepts for low- versus high-temperature origin of as yet underexplored Kiruna-type deposits.

## Methods

### Sampling

Samples from the Kiruna Mining District (*n* = 11) come from the type locality for Kiruna-type apatite-iron-oxide ores at Kiirunavaara, Sweden, as well as from other apatite-iron-oxide deposits in the district, including magnetite dykes in the footwall of the smaller deposit at Luossavaara, as well as massive ore from the Mertainen and Rektorn deposits. Mineralized samples from the Grängesberg Mining District (GMD) were collected from three drillcores (DC), DC 690 (*n* = 7), DC 717 (*n* = 3), and DC 575 (*n* = 3) that transect the deposit with a shallow plunge (<20 °) and were drilled at 650 m (*n* = 2) and 570 m below the surface respectively. In addition to massive apatite-iron-oxide samples, two Grängesberg samples were selected from magnetite veins and disseminations in the host rocks. One sample was collected from the smaller Blötberget apatite-iron-oxide deposit within the greater Grängesberg area and another from the nearby but contrasting marble-hosted hydrothermal/low-temperature iron oxide deposit at Björnberget (*n* = 1). Samples from El Laco (*n* = 6) were sampled at the surface and come from Laco Sur, the southern deposit in the area. To obtain a meaningful and widely applicable comparison of the apatite-iron-oxide ores with hydrothermal and magmatic reference samples, oxygen, and iron isotope values were also determined on magnetite from massive ore from the iron oxide-polymetallic skarn deposit at Dannemora in Sweden (*n* = 4), the banded iron formation at Striberg in Sweden (*n* = 1), the layered igneous intrusion of Panzhihua in China (*n* = 2), the Bushveld igneous complex in South Africa (*n* = 1), the Swedish layered igneous intrusions of Taberg (*n* = 1), Ulvön (*n* = 1), and Ruoutevare (*n* = 1), and from a gabbro bomb from Skjaldbreiður in Iceland (*n* = 1). Samples representative of recent igneous magnetites were chosen from basaltic andesites from Indonesia (*n* = 6), basalts, and dolerite from the Canary Islands (*n* = 3), dacites from New Zealand (*n* = 2), and a dolerite from Troodos massif in Cyprus (*n* = 1). An overview of the samples used in this study and details on their mineral assemblage and provenance is given in Supplementary Table 1. An outline of the geological setting of our sample suite is given in the Supplementary Information.

### Iron isotope analysis

Analysis of the individual magnetite samples for Fe isotopes was dominantly carried out at the Victoria University in Wellington, New Zealand (*n* = 47). The crystals were digested and chemically purified with concentrated HF and HNO_3_ acid and the analysis was then done using a ^57^Fe– ^58^Fe double spike and a Nu Plasma MC-ICP-MS (Multicollector-Inductively Coupled Plasma Mass Spectrometer). As a standard the international IRMM-014 CRM material was used. Full details on the Fe isotope analysis are given in Millet et al.^64^. All iron isotope data were recorded as δ^56^Fe, which is the deviation of ^56^Fe/^54^Fe relative to the IRMM-014 CRM standard material. The average 2*σ* error during iron isotope analysis was 0.03‰.

A set of magnetite samples (*n* = 11) was analysed for iron-isotopes by ALS Scandinavia Ltd. in Luleå, Sweden. The magnetite samples were prepared for analysis by microwave-assisted digestion in a HNO_3_ + HCl + HF mixture according to the method described in Ingri et al.^65^. The isotope analysis was then carried out with a Thermo Scientific Neptune MC-ICP mass spectrometer. The δ^56^Fe-values were calculated with relation to the IRMM-014 CRM standard. The 2*σ* error was calculated from two independent consecutive measurements and was on average also about 0.03‰. Six further magnetite samples were analyzed at the Vegacenter at the Swedish Museum of Natural History in Stockholm. The crystals were digested and chemically purified using concentrated HF and HNO_3_ and 10 M HCl acid following the procedures of Borrok et al.^66^ and Millet et al.^64^. The samples were diluted with 0.3 M HNO_3_ to a concentration of 2–3 ppm before measurement. The Fe isotope analyses were performed on a Nu Plasma II HR-MC-ICP-MS in pseudo-high-resolution mode to resolve interfering species. The samples were corrected for mass bias using the standard-sample bracketing technique, normalizing to the IRMM-014 standard. The average 2*σ* external reproducibility for the samples was 0.06‰ for δ^56^Fe.

### Oxygen isotope analysis

The analysis for oxygen isotopes was carried out at the University of Cape Town (South Africa) using a Finnigan DeltaXP dual inlet gas source mass spectrometer (*n* = 59). For the oxygen analysis the magnetite samples were prepared by laser fluorination^67^, whereby they were reacted with 10 kPa of BrF_5_, and the purified O_2_ was collected onto a 5 Å molecular sieve in a glass storage bottle. As a reference and calibration standard Monastery garnet was used^68^. All oxygen data were recorded in the usual δ^18^O notation relative to SMOW where δ^18^O = (*R*_sample_/*R*_standard _− 1)×1000, and *R *= the measured ratio ^18^O/^16^O. All oxygen isotope data were obtained with a 2*σ* error of ≤0.2‰.

## Supplementary information

Supplementary Information

Source Data

## Data Availability

The authors declare that all relevant data are available within the article and its supplementary information files.
