# Supplementary material for: Global Fe–O isotope correlation reveals magmatic origin of Kiruna-type apatite-iron-oxide ores
Source: Nat Commun. 2019 Apr 12;10:1712. doi: 10.1038/s41467-019-09244-4 (PMC6461606; doi:10.1038/s41467-019-09244-4)
Supplement: Supplementary file 1 — Supplementary Information [file 41467_2019_9244_MOESM1_ESM.pdf]

Supplementary Information

Supplementary Note 1: Sampling sites

1. Sampling locations for apatite-iron-oxide ores

1.1 Kiruna district, northernmost Sweden: The Kiirunavaara, Luossavaara and Mertainen deposits in the Kiruna Mining District in Lappland, northern Sweden are hosted by trachyandesites and rhyodacitic ignimbrites and tuffs with an age of 1.89 to 1.87 Ga [3,19,71–74]. These volcanic rocks are underlain by older greenstones which are supposed to have formed in an extensional setting [75]. Deformation in the area is generally non-penetrative, dominated by local shearing and brittle tectonics and while regional metamorphism in greenschist facies has been invoked, a lower overprint has been suggested at Kiirunavaara and Luossavaara [76,77]. The apatite-iron oxide ores in the Kiruna district are dominated by magnetite, with iron contents of 50-70 % and up to c. 20 % apatite [25]. The deposits of the Kiruna district hold pre-mining reserves of more than 2 billion tons of ore [5]. The ore bodies have been interpreted to of primarily magmatic origin on the basis of geochemical and textural observations including nodular ore textures and oxygen isotopes [5,24,26,78–81].

1.2 Grängesberg/Blötberget, Bergslagen, Central Sweden: The apatite-iron-oxide deposit at Grängesberg and the smaller Blötberget deposit are dominated by magnetite with subordinate hematite and additionally both oxides occur as associated veins and disseminations in the immediate host rocks[1,82]. In the massive ores, bands of fine-grained fluorapatite with associated REE phosphates as well as variable amounts of silicates are characteristic [41]. The main deposit at Grängesberg, the so called “Export Field” consists of iron oxide ores in the ratio of approximately 80 % magnetite and 20 % hematite. Hematite-dominated parts occur mostly in the structural footwall and in the vicinity of crosscutting pegmatite dykes. Alteration zones in the host rocks right next to the mineralisation comprise disseminated and discrete phyllosilicate

(biotite, chlorite) and amphibole-rich assemblages (so-called *sköl*) with variable amounts of iron oxides and fluorapatite [83]. The mineralisation is stratiform and dips between 50° and 70° towards the south-east and could be followed for more than 900 m at the surface where its width ranges between 50 and 100 m [84,85]. The ore is hosted by metavolcanic rocks of andesitic to dacitic compositions that belong to the c. 1.91-1.87 Ga volcano-sedimentary succession of Bergslagen [40,86].

1.3 El Laco, Pico Laco, Northern Chile: The apatite-iron oxide ores of El Laco are situated in Northern Chile at the flank of the Pliocence Pico Laco volcanic complex [5]. The area around El Laco hosts seven different deposits distributed over 30 km^2 with a total amount of 500 Mt of high grade (~60% Fe) ore [5]. The ore consists mainly of magnetite, however, hematite is also present as an oxidation product. Fission track dating of apatite crystals within the El Laco ore gave an age estimation of 2.1± 0.1 Ma [87]. The host rocks of the deposit consist of typical subduction zone andesites and dacites which have been hydrothermally altered with alteration increasing at depth [5,88,89]. Although hydrothermal activity was associated with the ore formation [5,88], the ore at El Laco is supposed to have formed by dominantly magmatic processes, involving an iron oxide-rich magma and magmatic fluids [62]. Textural analysis indicates that the ore resembles intrusive and extrusive magmatic activity, such as lava flows, pyroclastics and dykes, where the lava flow deposits are notably dominated by hematite [5,88]. A magmatic origin is also supported by features of iron oxide (magnetite) lava bombs, *aa* and *pahoehoe* lavas as well as vesicle-like cavities in addition to geochemical data from oxygen isotope analysis [5,62]. However, a hydrothermal origin is put forward on the basis of oxygen isotopes [32] arguing that the surprising isotopic homogeneity of magnetite samples at El Laco (~ +4.0 ‰) could not result from magmatic processes as a wider range of values would be expected due to magma cooling and hydrothermal processes associated with volcanic activity. Instead, these authors propose an origin by hydrothermal replacement with some high-$\delta^{18}O$ hydrothermal fluid as transport agent

[27,30,32] possibly also in some sort of evaporitic pan. The samples for this study represent massive magnetite ore from the Laco Sur apatite-iron oxide ore deposit at El Laco.

1.4 Bafq, Central Iran:

The Bafq-Saghand metallogenic zone is located in the Kashmar-Kerman Tectonic Zone (KKTZ) in Central Iran and comprises about 34 recorded iron ore mineralizations with nearly ~1500 Mt ore with an average grade of 55% Fe [90,91]. Among these deposits, larger apatite-iron-oxide ores that are currently mined include Chadormalu, Choghart, Se-Chahun, Lakke Saih and Esfordi. Some deposits, such as Esfordi and Gazestan, have high-grade apatite mineralizations and represent important phosphorus resources.

The deposits show a spectrum of mineralization styles such as massive orebodies, metasomatic replacements, stockworks and veins. The dominant minerals are magnetite, apatite and actinolite. Although the main Fe mineral is magnetite, all gradations towards hematite (through martitization) occur [92].

Most of the iron ore bodies occur as dome-shaped discordant to concordant structures, which consist mostly of lenses or irregular masses of massive magnetite surrounded by ore breccia and disseminated magnetite in the host rocks. In some places irregular bodies of massive magnetite are enclosed by a stockwork of magnetite, actinolite and apatite veins. An important feature of these deposits is that they frequently display gradational contacts with their host rocks. Sharp contacts are generally restricted to structurally controlled zones [92]. The apatite-iron-oxide mineralizations in the Bafq-Saghand zone are hosted by dolomitic and rhyolitic rocks of Cambrian Volcano-Sedimentary Units (CVSU).

The geological setting of the KKTZ is linked to a major episode of late Neoproterozoic to Early Cambrian orogenic activity in an active continental-margin environment [43].

The Origin of low-Ti apatite-iron oxide deposits of the Bafq-Saghand area has long been a matter of debate. In this case, several models have been proposed for these deposits which

include i) carbonatitic magmatism [93–95], ii) liquid immiscibility [96], iii) magmatic [11,97], iv) alkaline magmatism [42], v) magmas of the Kiruna-type [98], vi) hydrothermal Kiruna-type [92,99–101], vii) banded iron formations [102,103] and viii) magmatic-hydrothermal [91].

2. Sampling locations for Layered Igneous Intrusion reference materials

2.1 Bushveld, South Africa: The magnetite sample in this study comes from the Rustenburg Layered Suite (RLS) of the Bushveld complex, which is an 8 km thick succession of layered mafic and ultramafic rocks with an age of about 2.1 Ga [104,105]. The RLS is divided up into the Lower, Critical, Main and Upper zone, with the Critical Zone being the economically most important one since it holds the world's largest chromite and platinum-group element deposits [106]. However, magnetite is mined within the Upper Zone, which contains about 20 m in total thickness of pure magnetite in the form of several magnetite layers within 2 km thick magnetite-bearing gabbroic rocks [107]. The magnetite layers vary in thickness between 0.1 and 10 m and contain some silicates, mostly plagioclase feldspar [105]. The most prominent layer is called the Main Magnetite Layer from which the magnetite used in this study originates. The magnetite layers in the Upper zone are of magmatic origin and are assumed to have been formed by cycles of magma mixing of different FeO-rich magmas and subsequent cumulate emplacement [108].

2.2 Panzhihua, Sichuan Province, China: The layered igneous intrusion of Panzhihua is located in the Panxi Mining District, Sichuan Province, in South West China and is part of the Emeishan Large Igneous Province. It is a relatively unmetamorphosed and undeformed, 2 km thick, sill-like gabbroic intrusion which dips about 50°-60° towards the NW and extends about 19 km from NE to SW [109]. The intrusion is concordantly emplaced within late Neoproterozoic dolomite limestones, Permian syenites and Triassic shales and coal measures and is itself 263 Ma old [109]. The intrusion is divided into four zones based on differences in internal structure

and iron oxide mineralizations. These four zones are the marginal, lower, middle and upper zone. Iron ore occurs in the lower and middle zones. The mineralizations consist of both, massive lens-shaped or tabular ore bodies up to 60 m in thickness as well as disseminated ore. The massive ore bodies consist of >80% Ti-magnetite with variable amounts of clinopyroxene, plagioclase, and olivine. The average ore grade comprises 43 wt % FeO, 11.68 wt % TiO_2, and 0.30 wt % V_2O_5 [109]. On the basis of texture (e.g. vesicles), geochemistry and the absence of evidence for a skarn origin, the mineralization is interpreted to have formed from an oxide enriched melt [109]. The Panzhihua deposit is currently mined and holds a reserve of 1333 Mt of ore [109]. The two Panzhihua samples of this study represent massive Ti-magnetite ore.

2.3 Ruoutevare, Norrbotten, Sweden: The geology of the Ruoutevare area in Norrbotten, northern Sweden, comprises ultrabasic rock types such as peridotite and pyroxenite, which are associated with anorthosite and gabbro of Precambrian age [110,111]. Associated with the gabbro intrusion is a deposit of iron ore in the form of Ti-bearing magnetite layers with a grade of 54.2 wt. % FeO and 11 wt. % TiO_2 [112,113].

2.4 Taberg mine, Småland, Sweden: The Fe-Ti mineralization at Taberg is located in southern Sweden, about 12 km to the south of Lake Vättern, within the Protogine Zone, a 1.2 Ga old, 20 km wide and several 100 km long belt of ductile and brittle deformation [114–116]. The ore deposit consists of 1.2 Ga old troctolites (e.g. olivine gabbro) with high contents of Ti-rich magnetite ("titanomagnetite") and which have been affected by late Sveconorwegian amphibolite facies metamorphism [114,116]. The ore body has a dimension of c. 1 x 0.4 km and is hosted by an amphibolitised gabbro-dolerite, which has intruded the surrounding Småland granites [114,117]. The ore holds between 26 and 35 % "titanomagnetite" with a content of 28.7–32.3 % FeO [114]. Within the ore are plagioclase-rich layers which give the appearance of a layered igneous intrusion [114,117]. The ore is supposed to have been formed as a magmatic cumulate which

resulted from gravitational settling within a gabbroic magma [114,116]. The sample in this study is a massive Ti-magneite ore from the mine at Taberg.

2.5 Ulvön, Ångermanland, Central Sweden: The mineral ulvöspinel derives its name from the Ulvö island, where the layered igneous Ulvö Gabbro Complex is found [118] at the east coast of central Sweden. The intrusion consists of several gently dipping lopoliths, 30-80 km in diameter and 250-300 m in thickness [119,120]. These gabbroic lopoliths contain alternate bands of mafic and more felsic layers with thicknesses between 0.5 cm and 1 m, likely a result of magmatic cumulus processes [121]. These rocks are ~1.25 Ga old and have not been affected by regional metamorphic overprint or deformation [120,121]. Ti-magnetite occurs in distinct layers, like for example in the rhythmically layered zone of Norra Ulvön, which contains up to 10 cm thick bands with >50 % Fe-Ti oxides [121,122]. Such layers have also been mined for their metals [121]. Other common minerals in the Ulvö Gabbro Complex are plagioclase (labradorite), olivine, and clinopyroxene [119,121]. The sample used in this study is a massive Ti-magnetite ore from Norra Ulvön.

3. Sampling locations for volcanic reference materials

3.1 Canary Islands: Tenerife, located in the centre of the Canary archipelago, is the largest (2058 km^2) and highest (3718 m) island of the island group, which is situated over the Canary hot spot [123,124]. Volcanic activity on the island dates back to >6.5 Ma and is today seen in several small volcanoes and the Teide-Pico Viejo edifice [124]. Samples for this study originate from dykes in the NE rift zone of the island. The samples comprise ankaramites and basanites [125,126].

3.2 Cyprus: The island of Cyprus is located in a zone of underthrusting in the eastern part of the Mediterranean Sea, where the African plate is being pushed into the Eurasian plate. It can be divided into five more or less parallel belts, which trend approximately eastwards and are

convex towards the south [127]. Four of these belts are dominated by sedimentary rocks, most commonly limestones and loose sediments ranging in age from the Triassic to recent [127,128]. The fifth belt, the Troodos igneous massif, is dominated by mafic and ultramafic igneous rocks and represents an ophiolite sequence obducted during the Alpine orogeny [127,129]. The Troodos massif is about 11 km thick and divided up into basic and ultrabasic rocks in the center, the sheeted intrusive complex and the peripheral pillow lavas [127]. The rocks have been affected by metamorphism represented by diabase and serpentinite [127]. The sample used in this study comes from a dolerite dyke near Agros in the central part of the Troodos complex [130].

3.3 Iceland: The volcanic island of Iceland is located directly over the point in the North Atlantic, where asthenospheric flow interacts with a deep seated mantle plume [131], whose current plume channel lies beneath the Vatnajökull glacier and represents the surface expression of the Mid-Atlantic ridge [132]. Extensive volcanism is common on the island with more than 18 active volcanoes, which are often associated with rift zones and their volcanic fissure swarms [131]. Volcanic eruptions, often of explosive nature due to lava-snow interaction, occur every three to four years [133]. The main eruption products are tholeiitic basalts as well as basaltic andesites [134]. The magnetite sample used in this study comes from a basaltic lava bomb erupted from the Skjaldbreiður volcano in SW Iceland.

3.4 Indonesia: The investigated magnetite samples come from the Anak Krakatau [135,136], Agung [137], Gede [138], Kelut [139] and Merapi [140] volcanoes on the Indonesian Islands of Java and Bali. These are part of the Western Sunda-Banda arc, which developed during the Cenozoic through subduction of the Indian-Australian plate under the Eurasian plate [141,142]. Calc-alkaline volcanism with dacites and andesites are the typical eruption products in most recent times [142,143]. Beside volcanic rocks, the area also hosts several gold, tin and copper deposits associated with subduction zone volcanism [142].

3.5 New Zealand: Mount Ruapehu is a 2797 m high stratovolcano at the southern end of the Taupo Volcanic Zone (TVZ) on the North Island of New Zealand [144]. It is the largest currently active volcano on the Northern Island with the most recent eruption in 2007 and several other eruptive events during the last hundred years [145,146]. Volcanic activity in the TVZ is associated with the subduction of the Pacific Plate beneath the Australian Plate along the Hikurangi-Kermadec Trench system [147,148]. Mt. Ruapehu is underlain by Mesozoic meta-greywacke, which in turn is underlain by oceanic, metamorphosed igneous crust [146,149]. The typical eruption products are subduction zone andesites and dacites with porphyritic textures [150,151]. The magnetite content of the volcanic rocks at Mt. Ruapehu varies between less than 1 % and up to 6 % and is just over 1% on average [146]. Samples used in this study are two dacite rocks that come from the southern flank of Mt. Ruapehu.

4. Sampling sites for low-temperature hydrothermal ore deposit reference materials

4.1 Björnberget mines, Grängesberg Mining District, Bergslagen, Central Sweden: The Björnberget mines are located c. 3 km east-southeast of the Grängesberg Export field in the northwestern part of the Bergslagen ore province, south central Sweden. The magnetite-dominated iron ores occur as in part carbonate-banded, skarn-associated types, which may locally progress into true skarn iron ores [152,153]. The major iron ore zones are northeast-striking, and variably, but steeply (to 80°) dipping towards the southeast [152]. The ore-bearing carbonates and skarns of the Björnberget mines are in turn hosted by c. 1.91-1.88 Ga old felsic metavolcanic rocks (rhyolitic to rhyodacitic in composition), which are variably altered, but of which more well-preserved types exhibit what can be interpreted as primary laminations [86,153]. Later regional metamorphism to amphibolite facies grade, as well as three stages of ductile deformation have affected the older rocks in the area, including the Björnberget ore and its host rocks [85,86].

4.2 Dannemora mine, Bergslagen, southcentral Sweden: The Dannemora skarn iron ore deposit is situated in the eastern part of the Bergslagen ore province. It consists of 25 iron ore bodies, which are typically enriched in manganese, and some smaller sulphide deposits, which are all hosted by c. 1.9 Ga old meta-volcanic and meta-sedimentary rocks. These include meta-dacites and meta-rhyolites as well as calcitic and dolomitic meta-limestones (marbles). The latter can locally exhibit preserved stromatolitic textures [154]. The metavolcanic rocks have been interpreted as pyroclastic flow and air-fall deposits which together with the limestones were deposited in open marine, lagoonal and terrestrial (subaerial) environments [154]. The dolomitic marbles at Dannemora are very dark-coloured due to a content of 5-30 % of fine magnetite [154,155]. The area has been affected by greenschist facies metamorphism during the Svecokarelian orogeny and deformed at least twice, leading to isoclinal folding [154,156]. The iron ore at Dannemora consists to a great extent of massive strata-bound magnetite ore dipping 65-70° to the west, within an east-south-east syncline, and has an iron content of between 30 and 50 % [154,155]. The formation of the ore is believed to relate to circulating metal-bearing hydrothermal fluids. These fluids altered silica-rich units in the area and formed the major, mineralised skarn units through extensive reactions with the pre-exisiting limestones [154]. In some cases fluid rock interaction and evaporation may have altered the fluid composition and led to enrichment of heavier elements and isotopes. The Dannemora samples used in this study are all calcite-bearing magnetite ores and come from various locations in the Dannemora mine (see **Supplementary Table 1**).

4.3 Striberg mine, Bergslagen, Central Sweden: Banded Iron Formations (BIF), such as the deposit at Striberg, are found in the Bergslagen ore province, and are hosted by the 1.91-1.88 Ga old metavolcanic rocks [86,157]. In the Striberg area, extensive banded iron formations occur associated with skarn iron ores, in a complexly folded and deformed succession. The succession

shows a main structural trend in a northwest-southeasterly direction, and with moderately steep (c. 45-60º) dips to the northeast [152]. The main Striberg deposit consists mainly of alternating quartz and hematite-rich layers, normally of 1-10 mm thickness, and the silica content varies between 18 % and 28 % [24,86,157]. Typical for BIF deposits, there is a dominance of hematite as the main Fe-bearing mineral, however, magnetite is also present as an alteration product of the latter, and in some BIF ore types at Striberg the hematite has been completely converted to magnetite [158]. The iron content of the deposits lies between 30 % and 55 % [86,157] .

Reference List Supplementary Information

71. Lundbohm, H. Sketch of the Geology of the Kiruna district. *Geol. Fören. Stockholm Förhan.* **32,** 751–788 (1910).

72. Romer, R. L., Martinsson, O. & Perdahl, J. A. Geochronology of the Kiruna iron ores and hydrothermal alterations. *Econ. Geol.* **89,** 1249–1261 (1994).

73. Martinsson, O., Billström, K., Broman, C., Weihed, P. & Wanhainen, C. Metallogeny of the Northern Norrbotten Ore Province, Northern Fennoscandian Shield with emphasis on IOCG and apatite-iron ore deposits. *Ore Geol. Rev.* **78**, 447-492 (2016).

74. Lundberg, B. & Smellie, J. A. T. Painirova and mertainen iron ores: Two deposits of the kiruna iron ore type in Northern Sweden. *Econ. Geol.* **74,** 1131–1152 (1979).

75. Lindblom, S., Broman, C. & Martinsson, O. Magmatic-hydrothermal fluids in the Pahtohavare Cu-Au deposit in greenstone at Kiruna, Sweden. *Miner. Depos.* **31**, 307-318 (1996).

76. Bergman, S., Kübler, L. & Martinsson, O. (2001) *Description of regional geological and geophysical maps of northern Norrbotten County (east of the Caledonian orogeny)*. Geological Survey of Sweden Ba 56, 110 pp.

77. Berglund, J. & Andersson, U. B. *Kinematic analysis of geological structures in Block 34, Kiirunavaara* (2013). LKAB Investigation 13-746, 48 p. + 2 Appendix, 29 p.

78. Geijer, P. *The iron ores of the Kiruna type*. Geological Survey of Sweden C 367, 39 p (1931).

79. Geijer, P. *The Rektor ore body at Kiruna*. Geological Survey of Sweden C 514, 18 p (1950).

80. Harlov, D. E. *et al.* Apatite – monazite relations in the Kiirunavaara magnetite – apatite ore, northern Sweden. *Chem. Geol.* **191,** 47–72 (2002).

81. Andersson, U.B. *Coeval iron oxide and silicate magmas; structural evidence for*

immiscibility and mingling at Kiirunavaara and Luossavaara, Sweden. 12th SGA Biennial Metting, Proceedings, Uppsala, Sweden 12-15/8. Geological Survey of Sweden, 1635-1638 (2013).

82. Jiao, J. *Petrography and geochemistry of a section through the Blötberget apatite-iron oxide deposit, Bergslagen, south central Sweden*. Master thesis, Uppsala University, Uppsala, Sweden, 72 p (2011).

83. Jonsson, E. *et al. Oxygen isotopes and geochemistry of Palaeoproterozoic Kiruna-type deposits in the Bergslagen province, central Sweden.* Abstract volume, SGA biennial meeting 2011, Antofagasta, Chile, 494–496 (2011).

84. Johansson, H. Die eisenerzführende Formation in der Gegend von Grängesberg. *Geol. Fören. Stockholm Förhan.* **31,** 239–410 (1910).

85. Högdahl, K., Troll, V. R., Persson-Nilsson, K. & Jonsson, E. *Structural evolution of the apatite-iron oxide deposit at Grängesberg, Bergslagen, Sweden.* In: E. Jonsson et al. (eds.), Mineral deposit research for a high tech world, 11th SGA Biennial Meeting, Proceedings, 1650-1653 (2013).

86. Stephens, M. B. *et al. Synthesis of the bedrock geology on the Bergslagen region, Fennoscandian Shield, south-central Sweden*. Geological Survey of Sweden Ba 58, 259 p (2009).

87. Maksaev, V., Gardeweg, M., Ramírez, C. F. & Zentilli, M. *Aplicación del método trazas de fisión (fission track) a la datación de cuerpos de magnetita de El Laco e Incahuasi en el altiplano de la Región de Antofagasta*. In: Congreso Geológico Chileno, No. 5, Actas 1: B1-B23. Santiago (1988).

88. Naranjo, J., Henrquez, F. & Nyström, J. Subvolcanic contact metasomatism at El Laco Volcanic Complex, Central Andes. *Andean Geol.* **37,** 110–120 (2010).

89. Velasco, F. & Tornos, F. Insights on the effects of the hydrothermal alteration in the El Laco magnetite deposit (Chile). *Macla* **16,** 210–211 (2012).

90. Torab, F. *Geochemistry and Metallogeny of Magnetite-Apatite Deposits of the Bafq Mining District, Central Iran*. Unpublished PhD Thesis, Clausthal University of Technology, Germany (2008).

91. Majidi, S. A., Lotfi, M., Emami, M. H. & Nezafati, N. The genesis of iron oxide-apatite (IOA) deposits: evidence from the geochemistry of apatite in Bafq-Saghand district, Central Iran. *Geosciences* **27,** 233–244 (2017).

92. Torab, F. M. & Lehmann, B. Magnetite-apatite deposits of the Bafq district, Central Iran: apatite geochemistry and monazite geochronology. *Mineral. Mag.* **71,** 347–363 (2007).

93. Förster, H. & Borumandi, H. Jungpräkambrische Magnetit-Lava und Magnetit-Tuffe aus dem Zentraliran. *Die Naturwissenschaften* **58,** 524–524 (1971).

94. Darvishzadeh, A. Investigation on Esfordi phosphate deposit. *Journal of Science, University of Tehran*, 2–24 (1983).

95. Samani, B. A. Metallogeny of the Precambrian in Iran. *Precambrian Res.* **39,** 85–106 (1988).

96. Förster, H. & Jafarzadeh, A. The Bafq mining district in central Iran - a highly mineralized Infracambrian volcanic field. *Econ. Geol.* **89,** 1697–1721 (1994).

97. Moore, F. & Modabberi, S. Origin of Choghart iron oxide deposit, Bafq mining district, Central Iran: new isotopic and geochemical evidence. *J. Sci. Islam. Repub. Iran* **14,** 259–270 (2003).

98. Williams, G. J. & Houshmandzadeh, A. (1966) *A petrological and genetic study of the Choghart iron ore body and the surrounding rocks*. Geological Survey of Iran, 18 p.

99. Daliran, F. Kiruna-type iron oxide-apatite ores and apatitites of the Bafq District, Iran, with an emphasis on the REE geochemistry of their apatites. In: Porter, T.M., Ed., *Hydrothermal Iron Oxide Copper-Gold and Related Deposits: A Global Perspective*, PGC Publishing, Adelaied, Vol. 1, 303-320 (2002).

100. Jami, M., Dunlop, A. C. & Cohen, D. R. Fluid inclusion and stable isotope study of the

Esfordi apatite-magnetite deposit, central Iran. *Econ. Geol.* **102,** 1111–1128 (2007).

101. Bonyadi, Z., Davidson, G. J., Mehrabi, B., Meffre, S. & Ghazban, F. Significance of apatite REE depletion and monazite inclusions in the brecciated Se-Chahun iron oxide-apatite deposit, Bafq district, Iran: Insights from paragenesis and geochemistry. *Chem. Geol.* **281,** 253–269 (2011).

102. Aftabi, A. et al. Fluid inclusion and stable isotope study of the Esfordi apaptite-magnetite deposit,central Iran – A discussion. *Econ. Geol.* **104,** 137–139 (2009).

103. Mohseni, S. & Aftabi, A. Comment on "Significance of apatite REE depletion and monazite inclusions in the brecciated Sehchahun iron oxide–apatite deposit, Bafq district, Iran: Insights from paragenesis and geochemistry" by Bonyadi, Z., Davidson,G.J., Mehrabi, B., Meffre, S., Ghazban, F [Chem. Geol. 281, 253–269]. *Chem. Geol.* **334,** 378–381 (2012).

104. Walraven, F., Armstrong, R. A. & Kruger, F. J. A chronostratigraphic framework for the north-central Kaapvaal craton, the Bushveld Complex and the Vredefort structure. *Tectonophysics* **171,** 23–48 (1990).

105. Harne, D. M. W. & Von Gruenewaldt, G.. Ore-forming processes in the upper part of the Bushveld complex, South Africa. *J. African Earth Sci.* **20**, 77-89 (1995).

106. Saager, R. *Metallische Rohstoffe von Antimon bis Zirkonium*. Bank Vontobel, Zürich, 176 p. (1984).

107. Cawthorn, R. & Molyneux, T. Vanadiferous magnetite deposits of the Bushveld Complex. In: *Mineral Deposits of Southern Africa* **2,** edited by Anhaeusser C.R. and Maske S., Geological Society of South Africa, Johannesburg, 1251-1266 (1986).

108. Irvine, T. N., & Sharpe, M. R. *Magma mixing and the origin of stratiform oxide ore zones in the Bushveld and Stillwater Complexes*, In: Metallogeny of Basic and Ultrabasic Rocks, edited by M. J. Gallagher, et al., pp. 183–198, Inst. of Min. Metall., London, U. K. (1986)

109. Zhou, M. F. *et al.* Geochemistry, petrogenesis and metallogenesis of the Panzhihua gabbroic layered intrusion and associated Fe-Ti-V Oxide deposits, sichuan province, SW China. *J. Petrol.* **46**, 2253-2280 (2005).

110. Peterson W. On the Iron Ore Field of Routivare in Norrbotten Lane. *Geol. Fören. Stockholm Förhan.* **15,** 45 p. (1893).

111. Stigh, J. *Ultramafiter i svenska delen av fjällkedjan – Möjligt slutförvaringsplats för radioaktivt avfall? Rapport till Svensk Kärnbränsleförsörjning AB*. Geological Survey of Sweden, 57 p (1982).

112. Landergren, S. *On the Geochemistry of Swedish iron ores and associated rocks – A study on iron-ore formation.* Geological Survey of Sweden C 469, 182 p (1948).

113. Fischer, R. P. *Vanadium Resources in Titaniferous Magnetite Deposits*. US Geological Survey Professional Paper 926-B, 9 p. (1975).

114. Sandecki, J. Mineralogical and genetical aspects of the Smålands Taberg Fe-Ti-V ore, Protogine Zone of southern Sweden. *GFF* **122,** 351–358 (2000).

115. Söderlund, P., Söderlund, U., Möller, C., Gorbatschev, R. & Rodhe, A. Petrology and ion microprobe U-Pb chronology applied to a metabasic intrusion in southern Sweden: A study on zircon formation during metamorphism and deformation. *Tectonics* **23**, TC5005 (2004).

116. Larsson, D. & Söderlund, U. Lu-Hf apatite geochronology of mafic cumulates: An example from a Fe-Ti mineralization at Sm??lands Taberg, southern Sweden. *Chem. Geol.* **224,** 201–211 (2005).

117. Hjelmqvist, S. *The titaniferous iron–ore deposit of Taberg in the South of Sweden*. Geological Survey of Sweden C 512, 55 p (1950).

118. Mogensen, F. A ferro-ortho-titanate ore from Södra Ulvön. *Geol. Fören. Stockholm Förhan.* **68,** 578–587 (1946).

119. Magnusson, K. Å. & Larson, S. Å. A palaeomagnetic investigation of the Ulvö dolerite,

Ångermanland, central Sweden. *Lithos* **10**, 205-211 (1977).

120. Lundqvist, T. *Beskrivning till berggrundskartan över Västernorrlands län (English summary).* Geological Survey of Sweden Ba 31, 429 p (1990).

121. Larson, S. Å, Hogmalm, K. J. & Meurer, W. P. Character and significance of spectacular layering features developed in the thin, alkali-basaltic sills of the Ulv?? Gabbro Complex, Sweden. *Mineral. Petrol.* **92,** 427–452 (2008).

122. Larson, S. Å. & Magnusson, K.-Å. *The magnetic and chemical character of Fe-Ti oxides in the Ulvö dolerite, central Sweden.* Geological Survey of Sweden C 723, 29 p (1976).

123. Carracedo, J. C. *et al.* Hotspot volcanism close to a passive continental margin: the Canary Islands. *Geol. Mag.* **135,** 591–604 (1998).

124. Ancochea, E. *et al.* Volcanic evolution of the island of Tenerife (Canary Islands) in the light of new K-Ar data. *J. Volcanol. Geotherm. Res.* **44,** 231–249 (1990).

125. Deegan, F. M. *et al.* Crustal versus source processes recorded in dykes from the Northeast volcanic rift zone of Tenerife, Canary Islands. *Chem. Geol.* **334,** 324–344 (2012).

126. Delcamp, A. *et al.* Dykes and structures of the NE rift of Tenerife, Canary Islands: A record of stabilisation and destabilisation of ocean island rift zones. *Bull. Volcanol.* **74,** 963–980 (2012).

127. Gass, I. G. & Masson-Smith, D. The Geology and Gravity Anomalies of the Troodos Massif, Cyprus. *Philosphical Trans. R. Soc. London* **255,** 417–467 (1963).

128. Henson, F. R. S., Browne, R. V. & McGinty, J. A synopsis of the stratigraphy and geological history of Cyprus. *Q. J. Geol. Soc.* **105,** 1–41 (1949).

129. Allerton, S. & Vine, F. J. Spreading evolution of the Troodos ophiolite, Cyprus. *Geology* **19,** 637–640 (1991).

130. Stillman, C. J. Some dyke-Gabbro relationships in the Troodos Ophiolite, Cyprus. In: Gibson, I. L. et al. (eds.) *Cyprus Crustal Study Project: initial report, hole CY-4.* Geological Survey of Canada, Ottawa, Ontario, Canada: 107–113 (1989).

131. Trønnes, R. G. *Geology and geodynamics of Iceland*. Unpub. Ms., University of Iceland (2002).

132. Wolfe, C. J., Bjarnason, I. Th., VanDecar, J. C. & Solomon, S. C. Seismic structure of the Iceland mantle plume. *Nature* **385,** 245–247 (1997).

133. Gudmundsson, M. T. *et al.* Eruptions of Eyjafjallajökull Volcano, Iceland. *Eos* **91,** 190-191 (2010).

134. Sæmundsson, K. Outline of the geology of Iceland. *Jokull* **29,** 11–28 (1979).

135. Dahren, B. *et al.* Magma plumbing beneath Anak Krakatau volcano, Indonesia: Evidence for multiple magma storage regions. *Contrib. Mineral. Petrol.* **163,** 631–651 (2012).

136. Gardner, M. F. *et al.* Crustal differentiation processes at Krakatau Volcano, Indonesia. *J. Petrol.* **54,** 150–182 (2013).

137. Self, S. & Rampino, M. R. The 1963-1964 eruption of Agung volcano (Bali, Indonesia). *Bull. Volcanol.* **74,** 1521–1536 (2012).

138. Handley, H. K., Macpherson, C. G. & Davidson, J. P. Geochemical and Sr-O isotopic constraints on magmatic differentiation at Gede Volcanic Complex, West Java, Indonesia. *Contrib. Mineral. Petrol.* **159**, 885-908 (2010).

139. Jeffery, A. J. *et al.* The pre-eruptive magma plumbing system of the 2007-2008 dome-forming eruption of Kelut volcano, East Java, Indonesia. *Contrib. Mineral. Petrol.* **166,** 275-308 (2013).

140. Chadwick, J. P. *et al.* Petrology and geochemistry of igneous inclusions in recent Merapi deposits: A window into the sub-volcanic plumbing system. *Contrib. Mineral. Petrol.* **165**, 259-282 (2013).

141. Carlile, J. C. & Mitchell, A. H. G. Magmatic arcs and associated gold and copper mineralization in Indonesia. *J. Geochem. Explor.* **50,** 91–142 (1994).

142. Marcoux, E. & Milési, J. P. Epithermal gold deposits in West Java, Indonesia: geology, age and crustal source. *J. Geochem. Explor.* **50,** 393–408 (1994).

143. Van Bemmelen, R. W. (1949) *The geology of Indonesia*. Government Printing Office, The Hague, 732 p.

144. Gamble, J. A., Price, R. C., Smith, I. E. M., McIntosh, W. C. & Dunbar, N. W. $^{40}Ar/^{39}Ar$ geochronology of magmatic activity, magma flux and hazards at Ruapehu volcano, Taupo Volcanic Zone, New Zealand. *J. Volcanol. Geotherm. Res.* **120,** 271–287 (2003).

145. Jolly, A. D., Sherburn, S., Jousset, P. & Kilgour, G. Eruption source processes derived from seismic and acoustic observations of the 25 September 2007 Ruapehu eruption-North Island, New Zealand. *J. Volcanol. Geotherm. Res.* **191,** 33–45 (2010).

146. Price, R. C. *et al.* The anatomy of an andesite volcano: A time-stratigraphic study of andesite petrogenesis and crustal evolution at Ruapehu Volcano, New Zealand. *J. Petrol.* **53**, 2139-2189 (2012).

147. Gamble, J. A. *et al.* A fifty year perspective of magmatic evolution on Ruapehu Volcano, New Zealand: Verification of open system behaviour in an arc volcano. *Earth Planet. Sci. Lett.* **170,** 301–314 (1999).

148. Stern, T. *et al.* Crust-mantle structure of the central North Island, New Zealand, based on seismological observations. *J. Volcanol. Geotherm. Res.* **190,** 58–74 (2010).

149. Price, R. C. *et al.* Crustal and mantle influences and U-Th-Ra disequilibrium in andesitic lavas of Ngauruhoe volcano, New Zealand. *Chem. Geol.* **277,** 355–373 (2010).

150. Graham, I. J. & Hackett, W. R. Petrology of calc-alkaline lavas from Ruapehu volcano and related vents, taupo volcanic zone, New Zealand. *J. Petrol.* **28,** 531–567 (1987).

151. Price, R. C. *et al.* An integrated model for the temporal evolution of andesites and rhyolites and crustal development in New Zealand's North Island. *J. Volcanol. Geotherm. Res.* **140,** 1-24 (2005).

152. Geijer, P. & Magnusson, N. H. *De mellansvenska järnmalmernas geologi*. Geological Survey of Sweden Ca 35, 654 p (1944).

153. Gavelin, S. Studier över berggrunden i Björnbergsfältet. *Geol. Fören. Stockholm Förhan.*

55, 455–498 (1933).

154. Lager, I. *The geology of the Palaeoproterozoic limestone-hosted Dannemora iron deposit, Sweden*. Sveriges Geologiska Undersökning Rapporter och meddelanden 107, 49 p (2001).

155. Lager, I. The Dannemora iron ore deposit. In: Lundström, I. & Papunen, H. (eds.): *Mineral deposits of southwestern Finland and the Bergslagen province, Sweden. 7th IAGOD symposium and Nordkalott project meeting, excursion guide No. 3*. Geological Survey of Sweden Ca 61, 26–30 (1986).

156. Dahlin, P., Allen, R. & Sjöström, H. Palaeoproterozoic metavolcanic and metasedimentary succession hosting the Dannemora iron ore deposits, Bergslagen region, Sweden. *GFF* **134,** 71-85 (2012).

157. Allen, R., Ripa, M. & Jansson, N. *Palaeoproterozoic volcanic- and limestonehosted Zn-Pb-Ag-(Cu-Au) massive sulphide deposits and Fe oxide deposits in Bergslagen, Sweden*. IGC Excursion Nr. 12 (August 2008): 16-21 (2008).

158. Magnusson, N.H. *The origin of the iron ores in central Sweden and the history of their alterations.* Geological Survey of Sweden C 643, 127 p (1970).

159. Lundh J. *A lithogeochemical study of Northern Sweden and the Kiruna and Malmberget iron-apatite ore deposits*. Master thesis, Uppsala University, 106 p (2014).

160. Zhao, Z. F. & Zheng, Y. F. Calculation of oxygen isotope fractionation in magmatic rocks. *Chem. Geol.* **193,** 59–80 (2003).

161. Zheng, Y. Calculation of oxygen isotope fractionation in metal oxides. *Chem. Geol.* **55,** 2299–2307 (1991).

162. Hoefs, J. *Stable isotope geochemistry*. Springer, Berlin, 286 p (1997).

163. Jolis, E. M. (2013) *Magma-Crust Interaction at Subduction Zone Volcanoes,* Doctoral dissertation Acta Universitatis Upsaliensis, Uppsala, Sweden, 244 p.

164. Dauphas, N. & Rouxel, O. Mass spectrometry and natural variations of iron isotopes.

Mass Spectrom. Rev. **25,** 515–520 (2006).

165. Hou, Z. *et al.* Geology, fluid inclusions, and oxygen isotope geochemistry of the Baiyinchang pipe-style volcanic-hosted massive sulfide Cu deposit in Gansu Province, Northwestern China. *Econ. Geol.* **103**, 269-292 (2008).

166. Rieger, A. A., Marschik, R. & Díaz, M. The evolution of the hydrothermal IOCG system in the Mantoverde district, northern Chile: New evidence from microthermometry and stable isotope geochemistry. *Miner. Depos.* **47**, 359-369 (2012).

167. Beard, B. L. *et al.* Application of Fe isotopes to tracing the geochemical and biological cycling of Fe. *Chem. Geol.* **195**, 87-117 (2003).

168. Beard, B. L. & Johnson, C. M. Fe Isotope Variations in the Modern and Ancient Earth and Other Planetary Bodies. *Rev. Mineral. Geochem.* **55,** 319–357 (2004).

Supplementary Tables

Supplementary Table 1. Oxygen and iron isotope analysis for magnetite from apatite-iron oxide ores and reference materials

Sample	Sample Description	Sample Provenance	$\delta^{18}O$ in ‰	2σ	$\delta^{56}Fe$ in ‰	2σ
**Apatite-iron oxide ore**						
**Kiruna Mining District (KMD), Northern Sweden**						
Kiruna-17NY28	Banded massive (Ap-)magnetite ore	Kiirunavaara mine, Kiruna	4.1	±0.2	0.19	±0.03
K-Mt-1 (907/75)	Massive magnetite ore	Kiirunavaara mine, Kiruna	-1.0	±0.2	0.20	±0.03
Ki-Mi-2a (1079/251)	Massive magnetite ore	Kiirunavaara mine, Kiruna	0.1	±0.2	0.21	±0.02
Ki-Mi-2b (1079/251)	Massive magnetite ore	Kiirunavaara mine, Kiruna	-0.7	±0.2	0.22	±0.04
K-Mt-1079/303	Massive magnetite ore	Kiirunavaara mine, Kiruna	-0.3	±0.2	0.27	±0.04
K-Mt-1079/437	Massive magnetite ore	Kiirunavaara mine, Kiruna	0.6	±0.2	0.16	±0.02
M1931*	Massive magnetite ore	Kiirunavaara mine, Kiruna	0.1	±0.2	-	-
M1937*	Skeletal magnetite ore	Kiirunavaara mine, Kiruna	1.4	±0.2	-	-
LVA-3	Massive magnetite ore	Luossavaara mine, Kiruna	1.2	±0.2	0.23	±0.02
LVA-FW-1	Massive magnetite ore	Luossavaara mine, Kiruna	1.4	±0.2	0.12	±0.04
LVA-FW-2	Massive magnetite ore	Luossavaara mine, Kiruna	1.2	±0.2	0.27	±0.03
K-Mt-3	Massive magnetite ore	Mertainen mine, Kiruna	1.9	±0.2	0.41	±0.03
K-Mt-4	Massive magnetite ore	Mertainen mine, Kiruna	1.6	±0.2	0.29	±0.03
M7557*	Massive magnetite ore	Rektorn mine, Kiruna	2.5	±0.2	-	-
**Grängesberg Mining District (GMD), Central Sweden**						
DC717-KES090068	Massive (Ap-)magnetite ore	Grängesberg mine, Grängesberg	1.2	±0.2	0.40	±0.03
DC717-KES090070	Massive (Ap-)magnetite ore	Grängesberg mine, Grängesberg	1.8	±0.2	0.24	±0.03
DC717-KES090072	Massive (Ap-)magnetite ore	Grängesberg mine, Grängesberg	0.9	±0.2	0.33	±0.03
DC717-KES090084	Magnetite vein in intermediate volcanic rock	Grängesberg mine, Grängesberg	-1.1	±0.2	0.11	±0.03
DC690-KES090011	Massive (Ap-)magnetite ore	Grängesberg mine, Grängesberg	2.8	±0.2	0.31	±0.03
DC690-KES090012	Massive (Ap-)magnetite ore	Grängesberg mine, Grängesberg	1.2	±0.2	0.31	±0.04
DC690-KES090020	Massive Ap-veined magnetite ore	Grängesberg mine, Grängesberg	1.1	±0.2	0.30	±0.04
DC690-KES090024	Massive (Ap-)magnetite ore	Grängesberg mine, Grängesberg	1.0	±0.2	0.26	±0.03
DC690-KES090027	Ap-veined/banded massive magnetite ore	Grängesberg mine, Grängesberg	1.2	±0.2	0.29	±0.03
DC690-KES090030	Silicate-spotted massive (Ap-)magnetite ore	Grängesberg mine, Grängesberg	1.8	±0.2	0.39	±0.04
DC690-KES090034	Coarse-grained Ap-spotted massive magnetite ore	Grängesberg mine, Grängesberg	0.5	±0.2	0.27	±0.03
DC690-KES090044	Disseminated magnetite in intermediate volcanic rock	Grängesberg mine, Grängesberg	-1.0	±0.2	0.24	±0.03
DC575-KES103011	Magnetite-dominated massive ore	Grängesberg mine, Grängesberg	1.8	±0.2	0.31	±0.03
DC575-KES103016	Coarse, massive (Ap-)magnetite ore	Grängesberg mine, Grängesberg	1.5	±0.2	0.27	±0.04
DC575-KES103003	Magnetite-dominated massive ore	Grängesberg mine, Grängesberg	0.2	±0.2	1.0	±0.03
KES091013b	Massive (Ap-)magnetite ore	Blötberget mine, Blötberget	0.1	±0.2	0.33	±0.03
**El Laco Ap-Fe-oxide deposit, Chile**						
EJ-LS-11-1	Massive magnetite ore	Laco Sur, El Laco	1.9	±0.2	0.28	±0.03
EJ-LS-11-2	Massive magnetite ore	Laco Sur, El Laco	-4.3	±0.2	0.24	±0.03
EJ-LS-11-3	Massive magnetite ore	Laco Sur, El Laco	-1.9	±0.2	0.36	±0.03
EJ-LS-11-4	Massive magnetite ore	Laco Sur, El Laco	4.2	±0.2	0.34	±0.03
LS-2	Massive magnetite ore	Laco Sur, El Laco[1]	4.3	±0.2	0.27	±0.03
LS-52	Massive magnetite ore	Laco Sur, El Laco[1]	4.4	±0.2	0.28	±0.03

Bafq Mining District, Iran

13.C.88	Massive magnetite ore	Sechahun, Bafq	2.4	±0.2	0.20	±0.06
13.C.106	Massive magnetite ore	Lakke Siah, Bafq	2.3	±0.2	0.26	±0.05
13.C.216	Massive magnetite ore	Chadormalu, Bafq	0.6	±0.2	0.24	±0.03
13.C.217	Massive magnetite ore	Chadormalu, Bafq	0.6	±0.2	0.32	±0.02
13.C.219	Massive magnetite ore	Chadormalu, Bafq	2.8	±0.2	0.27	±0.07
14.22	Massive magnetite ore	Esfordi, Bafq	3.4	±0.2	0.32	±0.01

Plutonic reference material

Ruoutevare	Ti-magnetite, layered igneous intrusion	Kvikjokk, Norrbotten, Sweden[2]	3.2	±0.2	0.31	±0.03
Ulvön	Ti-magnetite, layered igneous intrusion	Ulvön island, Ångermanland, Sweden[2]	4.0	±0.2	0.13	±0.03
Taberg	Ti-magnetite, layered igneous intrusion	Iron mine, Taberg, Småland, Sweden[3]	4.1	±0.2	0.23	±0.04
EM419	Massive Fe-Ti magnetite ore	Northern pit, Panzhihua, China[4]	4.8	±0.2	0.61	±0.05
EM424	Massive Fe-Ti magnetite ore	Nalahe, Panzhihua, China[4]	2.8	±0.2	0.12	±0.04
Bushveld	Massive magnetite ore	Upper Zone, Bushveld Complex, South Africa[4]	1.8	±0.2	-	-
Gabbrobomb	Magnetite from a gabbro xenolith	NW-Flank, Skjaldbreiður, Iceland[3]	4.6	±0.2	0.46	±0.03

Volcanic reference material

TEF-NER-18	Magnetite from an ankaramite dyke	NE Rift Zone, Tenerife, Spain	-	-	0.07	±0.05
TEF-NER-57B	Magnetite from an ankaramite dyke	NE Rift Zone, Tenerife, Spain	3.7	±0.2	0.16	±0.02
TEF-NER-70	Magnetite from a pyroxene phyric dyke	NE Rift Zone, Tenerife, Spain	3.7	±0.2	0.10	±0.02
MG-07	Igneous magnetite from dacite	S-Flank, Mt. Ruapehu, New Zealand[5]	-	-	0.32	±0.03
MG-09	Igneous magnetite from dacite	S-Flank, Mt. Ruapehu, New Zealand[5]	-	-	0.29	±0.03
Kelut A1	Igneous magnetite from basaltic andesite	Mt. Kelut, Java, Indonesia	-	-	0.10	±0.04
GD-D-2	Igneous magnetite from basaltic andesite	Gede Dome, Java, Indonesia	-	-	0.12	±0.03
AK-B1	Igneous magnetite from basaltic andesite	SE-Flank, Anak Krakatau, Indonesia	-	-	0.06	±0.03
AK-B3	Igneous magnetite from basaltic andesite	SE-Flank, Anak Krakatau, Indonesia	-	-	0.16	±0.03
A-BA-1	Igneous magnetite from basaltic andesite	Mt. Agung, Bali, Indonesia	-	-	0.18	±0.05
M-BA06-KA-3	Igneous magnetite from basaltic andesite	Mt. Merapi, Java, Indonesia	3.9	±0.2	0.17	±0.03
83/CRS/6	Igneous magnetite from a dolerite dyke	Agros, Troodos Massif, Cyprus[6]	-	-	0.34	±0.03

Low-temperature or hydrothermal magnetites

KES091007B	Calcite bearing-magnetite ore	Björnberget, Sweden	-0.8	±0.2	-0.02	±0.03
DM-1	Iron-skarn magnetite ore	Botenhäll, Dannemora, Sweden[7]	-0.4	±0.2	-0.36	±0.03
DM-2	Iron-skarn magnetite ore	Norrnäs 3, Dannemora, Sweden[7]	-0.7	±0.2	0.01	±0.03
DM-3	Iron-skarn magnetite ore	Konstäng, Dannemora, Sweden[7]	2.1	±0.2	-0.43	±0.03
DM-4	Iron-skarn magnetite ore	Strömsmalmen, Dannemora, Sweden[7]	-0.6	±0.2	-0.35	±0.03
EJ092008	Magnetite from a banded iron formation deposit	Striberg, Bergslagen, Sweden	-1.2	±0.2	-0.57	±0.03

* Data from Lundh (2014) (ref.[159])

1. Samples donated by Dr. Jan-Olov Nyström, Naturhistoriska Riksmuseet, Stockholm, Sweden
2. Samples from the sample collection of the Geological Survey of Sweden, Uppsala, Sweden
3. Sample from the sample collection, Department of Earth Science, Uppsala University, Sweden
4. Samples collected and donated by Prof. Nicholas Arndt, Université Joseph Fourier, Grenoble, France
5. Samples donated by Prof. John Gamble, Department of Geology, Victoria University of Wellington, New Zealand
6. Samples donated by Prof. Christopher J. Stillman, Department of Geology, Trinity College Dublin, Ireland
7. Samples donated by Gunnar Rauseus, Dannemora Mineral AB, Österbybruk, Sweden

Supplementary Table 1. Oxygen and iron isotope analysis for magnetite from apatite-iron oxide ores and reference materials

Supplementary Table 2. Results for the magma (900°C)/magmatic water (800°C) equilibrium re-calculation

Sample	Rock type	$\delta^{18}O$	$\delta^{56}Fe$	$\delta^{18}O$ andesite	Value fit	$\delta^{18}O$ dacite	Value fit	$\delta^{18}O$ water	Value fit	$\delta^{56}Fe$ water	$\delta^{56}Fe$ magma
Kiruna	Massive ore	4.1	0.19	8.1	✓	8.4	✓	9.3	✓	-0.05	0.16
K-Mt-1	Massive ore	-1.0	0.20	3.0	X	3.4	X	4.3	X	-0.04	0.17
Ki-Mi-2a	Massive ore	0.1	0.21	4.0	X	4.4	X	5.2	✓	-0.04	0.18
Ki-Mi-2b	Massive ore	-0.7	0.22	3.3	X	3.6	X	4.5	X	-0.02	0.19
K-Mt-1079/303	Massive ore	-0.3	0.27	3.6	X	4.0	X	4.9	X	0.03	0.24
K-Mt-1079/437	Massive ore	0.6	0.16	4.6	X	4.9	X	5.8	✓	-0.08	0.13
M1931	Massive ore	0.1	-	4.1	X	4.4	X	5.3	✓	-	-
M1937	Massive ore	1.4	-	5.4	X	5.7	✓	6.6	✓	-	-
LVA-3	Massive ore	1.2	0.23	5.2	X	5.5	X	6.4	✓	-0.02	0.19
LVA-FW-1	Massive ore	1.4	0.12	5.4	X	5.7	✓	6.6	✓	-0.12	0.09
LVA-FW-2	Massive ore	1.2	0.27	5.1	X	5.5	X	6.4	✓	0.03	0.24
K-Mt-3	Massive ore	1.9	0.41	5.9	✓	6.2	✓	7.1	✓	0.17	0.38
K-Mt-4	Massive ore	1.6	0.29	5.6	X	5.9	✓	6.8	✓	0.05	0.26
M7557	Massive ore	2.5	-	6.5	✓	6.8	✓	7.7	✓	-	-
DC717-KES090068	Massive ore	1.1	0.40	5.1	X	5.4	X	6.3	✓	0.16	0.37
DC717-KES090070	Massive ore	1.8	0.24	5.8	✓	6.1	✓	7.0	✓	-0.01	0.21
DC717-KES090072	Massive ore	0.9	0.33	4.8	X	5.2	X	6.1	✓	0.09	0.30
DC690-KES090011	Massive ore	2.8	0.31	6.7	✓	7.1	✓	8.0	✓	0.07	0.28
DC690-KES090012	Massive ore	1.2	0.31	5.2	X	5.5	X	6.4	✓	0.07	0.28
DC690-KES090020	Massive ore	1.1	0.30	5.0	X	5.4	X	6.3	✓	0.06	0.27
DC690-KES090024	Massive ore	1.0	0.26	5.0	X	5.3	X	6.2	✓	0.02	0.23
DC690-KES090027	Massive ore	1.2	0.29	5.2	X	5.5	X	6.4	✓	0.05	0.26
DC690-KES090030	Massive ore	1.8	0.39	5.8	✓	6.1	✓	7.0	✓	0.15	0.36
DC690-KES090034	Massive ore	0.5	0.27	4.5	X	4.8	X	5.7	✓	0.03	0.24
DC575-KES103003	Massive ore	1.8	0.31	5.7	✓	6.1	✓	7.0	✓	0.07	0.28
DC575-KES103011	Massive ore	1.5	0.27	5.5	X	5.8	X	6.7	✓	0.03	0.24
DC575-KES103016	Massive ore	0.1	0.33	4.1	X	4.4	X	5.3	✓	0.09	0.30
DC575-KES103003	Massive ore	0.2	1.0	4.2	X	4.5	X	5.4	✓	0.76	0.97
DC690-KES090044	Disseminated magnetite	-1.0	0.24	3.0	X	3.3	X	4.2	X	0.00	0.21
DC717-KES090084	Magnetite vein	-1.1	0.11	2.8	X	3.1	X	4.1	X	-0.13	0.08
EJ-LS-11-1	Massive ore	1.9	0.28	5.9	✓	6.2	✓	7.1	✓	0.04	0.25
EJ-LS-11-2	Massive ore	-4.3	0.24	-0.3	X	0.0	X	0.9	X	0.00	0.21
EJ-LS-11-3	Massive ore	-1.9	0.36	2.1	X	2.4	X	3.3	X	0.12	0.33
EJ-LS-11-4	Massive ore	4.2	0.34	8.1	✓	8.5	✓	9.4	✓	0.10	0.31
LS-2	Massive ore	4.3	0.27	8.2	✓	8.6	✓	9.5	✓	0.03	0.24
LS-52	Massive ore	4.4	0.28	8.4	✓	8.7	✓	9.6	✓	0.04	0.25
13.C.88	Massive ore	2.4	0.20	6.4	✓	6.7	✓	7.6	✓	-0.04	0.17
13.C.106	Massive ore	2.3	0.26	6.2	✓	6.6	✓	7.5	✓	0.02	0.23
13.C.216	Massive ore	0.6	0.24	4.5	X	4.9	X	5.8	✓	0.00	0.21

13.C.217	Massive ore	0.6	0.32	4.6	X	4.9	X	5.8	✓	0.08	0.29
13.C.219	Massive ore	2.8	0.27	6.8	✓	7.1	✓	8.0	✓	0.03	0.24
14.22	Massive ore	3.4	0.32	7.4	✓	7.7	✓	8.6	✓	0.08	0.29

Oxygen:

1. 1000lnα $_{(mt\text{-}basalt)}$ = -3.4 ‰; 1000lnα $_{(mt\text{-}andesite)}$ = -4.0 ‰; 1000lnα $_{(mt\text{-}dacite)}$ = -4.3 ‰; regular range of basalts, arc andesites/dacites +5.7 to +8 ‰ (ref.[57,58,160])
2. 1000lnα $_{(mt\text{-}water\ 800°C)}$ = -5.2 ‰; regular range for magmatic waters 5-10 ‰ (ref.[161,162])

Iron:

1. 1000lnα $_{(mt\text{-}magma)}$ = 0.03 ‰; regular range of arc andesites/dacites +0.00 to +0.12 ‰ (ref.[39])
2. 1000lnα $_{(mt\text{-}water\ 800°C)}$ = 0.24 ‰; regular range for magmatic waters 0.00 to -0.35‰ (ref.[39,70])

✓ = in equilibrium with magma/magmatic water ; X= not in equilibrium with common magmatic values

Supplementary Table 2. Results for the magma (900°C)/magmatic water (800°C) equilibrium re-calculation

Supplementary Table 3. Results for the magmatic water (625°C) equilibrium re-calculation

Sample	Rock type	$\delta^{18}O$	$\delta^{56}Fe$	$\delta^{18}O$ water	Value fit	$\delta^{56}Fe$ water
Ki-Mi-2a	Massive ore	0.1	0.21	6.3	✓	-0.14
K-Mt-1079/437	Massive ore	0.6	0.16	6.8	✓	-0.19
M1931	Massive ore	0.1	-	6.3	✓	
DC690-KES090034	Massive ore	0.5	0.27	6.7	✓	-0.08
DC575-KES103003	Massive ore	0.2	1.0	6.4	✓	0.65
DC575-KES103016	Massive ore	0.1	0.33	6.3	✓	-0.02
13.C.216	Massive ore	0.6	0.24	6.8	✓	-0.11
13.C.217	Massive ore	0.6	0.32	6.8	✓	-0.03

Oxygen: $1000\ln\alpha_{(\text{mt-water } 625°C)} = -6.2$ ‰ (ref.[161])
Iron: $1000\ln\alpha_{(\text{mt-water } 625°C)} = 0.35$ ‰ (ref.[39])
✓ = in equilibrium with magma/magmatic water ; X= not in equilibrium with common magmatic values (ref.[70,162])

Supplementary Table 3. Results for the magmatic water (625°C) equilibrium re-calculation

Supplementary Table 4. Results for the volcanic and plutonic reference material equilibrium re-calculation

Sample	Rock type	$\delta^{18}O$	$\delta^{56}Fe$	$\delta^{18}O$ basalt	$\delta^{56}Fe$ magma
TEF-NER-57B	Magnetite from an ankaramite dyke	3.7	0.16	7.1	0.13
TEF-NER-70	Magnetite from a pyroxene phyric dyke	3.7	0.10	7.1	0.07
M-BA06-KA-3	Igneous magnetite from basaltic andesite	3.9	0.17	6.2	0.14
TEF-NER-18	Magnetite from an ankaramite dyke	-	0.07	7.0	0.04
Kelut A1	Igneous magnetite from basaltic andesite	-	0.10	7.0	0.07
GD-D-2	Igneous magnetite from basaltic andesite	-	0.12	7.5	0.09
AK-B1	Igneous magnetite from basaltic andesite	-	0.06	6.4	0.03
AK-B3	Igneous magnetite from basaltic andesite	-	0.16	6.4	0.13
A-BA-1	Igneous magnetite from basaltic andesite	-	0.18	6.7	0.15
Gabbrobomb	Magnetite from a gabbro xenolith	4.6	0.46	8.0	0.43
Ruoutevare	Ti-magnetite, layered igneous intrusion deposit	3.2	0.31	6.6	0.28
Ulvön	Ti-magnetite, layered igneous intrusion deposit	3.9	0.13	7.4	0.10
Taberg	Ti-magnetite, layered igneous intrusion deposit	4.1	0.23	7.5	0.20
EM419	Massive Fe-Ti magnetite ore	4.8	0.61	8.3	0.58
EM424	Massive Fe-Ti magnetite ore	2.8	0.12	6.2	0.09

Oxygen: $1000\ln\alpha_{(mt\text{-}basalt)} = -3.4$ ‰; $1000\ln\alpha_{(mt\text{-}andesite)} = -4.0$ ‰; $1000\ln\alpha_{(mt\text{-}dacite)} = -4.3$ ‰; regular range of basalts, arc andesites/dacites +5.7 to +8 ‰ (ref.[57,58,160])

Iron: $1000\ln\alpha_{(mt\text{-}magma)} = 0.03$ ‰; regular range of arc andesites/dacites +0.00 to +0.12 ‰ (ref.[39])

For volcanic samples where no $\delta^{18}O$-value was obtained estimates for magma $\delta^{18}O$ were taken from Jolis (2013) (ref.[163]).

Supplementary Table 4. Results for the volcanic and plutonic reference material equilibrium re-calculation

Supplementary Table 5. Results for equilibrium re-calculation of literature data

Sample	$\delta^{18}O$	$\delta^{56}Fe$	$\delta^{18}O$ water	$\delta^{56}Fe$ water	$\delta^{18}O$ andesite	$\delta^{56}Fe$ magma
**Los Colorados, Chilean Iron Belt (Bilenker et al. 2016)**						
05–3.30	2.41	0.22	6.9	-0.13	6.4	0.19
05–20.7	3.04	0.09	7.5	-0.26	7.0	0.06
05–32	2.75	0.22	7.3	-0.13	6.8	0.19
05–52.2	3.17	0.14	7.7	-0.21	7.2	0.11
05–72.9	2.36	0.13	6.9	-0.22	6.4	0.1
05–82.6	2.76	0.08	7.3	-0.27	6.8	0.05
05–90	2.99	0.21	7.5	-0.14	7.0	0.18
05–106	2.78	0.12	7.3	-0.23	6.8	0.09
05–126.15	2.48	0.1	7.0	-0.25	6.5	0.07
04–38.8	2.04	0.18	6.5	-0.17	6.0	0.15
04–66.7	1.92	0.18	6.4	-0.17	5.9	0.15
04–129.3	2.62	0.22	7.1	-0.13	6.6	0.19
04–104.4	2.43	0.24	6.9	-0.11	6.4	0.21
**Pea Ridge and Pilot Knob (Childress et al. 2016)**						
PR18	2.12	0.35	8.3	0	4.9	0.32
PR-64A	4.87	0.2	11.1	-0.15	7.7	0.17
PR-77A	5.11	0.21	11.3	-0.14	7.9	0.18
PR-82A	5.9	0.1	12.1	-0.25	8.7	0.07
PR-82B	7.03	0.07	13.2	-0.28	9.8	0.04
PR-37	4.5	0.07	10.7	-0.28	7.3	0.04
PR-144	5.04	0.26	11.2	-0.09	7.8	0.23
PK-1145-965.8	6.68	0.19	12.9	-0.16	9.5	0.16
PK-1145-979.5	6.21	0.24	12.4	-0.11	9.0	0.21

Oxygen:
1. $1000\ln\alpha_{(mt\text{-}andesite)}$ = -4.0 ‰ (ref.[14]) or -2.8 ‰ (ref. [48])
2. $1000\ln\alpha_{(mt\text{-}water\ 625°C)}$ = -4.5 ‰ (ref. [14]) or -6.2 ‰ (ref. [48])

Iron:
1. $1000\ln\alpha_{(mt\text{-}magma)}$ = 0.03 ‰ (ref.[39])
2. $1000\ln\alpha_{(mt\text{-}water\ 625°C)}$ = 0.35 ‰ (ref.[39])

Supplementary Table 5. Results for equilibrium re-calculation of literature data

Supplementary Table 6. Results for hydrothermal fluid (375°C) equilibrium re-calculation

Sample	$\delta^{18}O$	$\delta^{56}Fe$	$\delta^{18}O$ fluid	$\delta^{56}Fe$ fluid
KES090044	-1.0	0.24	6.80	-0.26
KES090084	-1.1	0.11	6.70	-0.39
EJ-LS-11-2	-4.3	0.24	3.50	-0.26
EJ-LS-11-3	-1.9	0.36	5.90	-0.14
K-mt-1	-0.95	0.20	6.9	-0.30
Ki-mi-2b	-0.69	0.22	7.1	-0.28
K-mt-1079/303	-0.33	0.27	7.5	-0.23

Oxygen: $1000\ln\alpha_{\text{(mt-water 375°C)}}$ = -7.8 ‰ (ref.[161])
Iron: $1000\ln\alpha_{\text{(mt-water 375°C)}}$ = 0.5 ‰ (ref.[39])

Supplementary Table 6. Results for hydrothermal fluid (375°C) equilibrium re-calculation

Supplementary Figures

Supplementary Fig.1

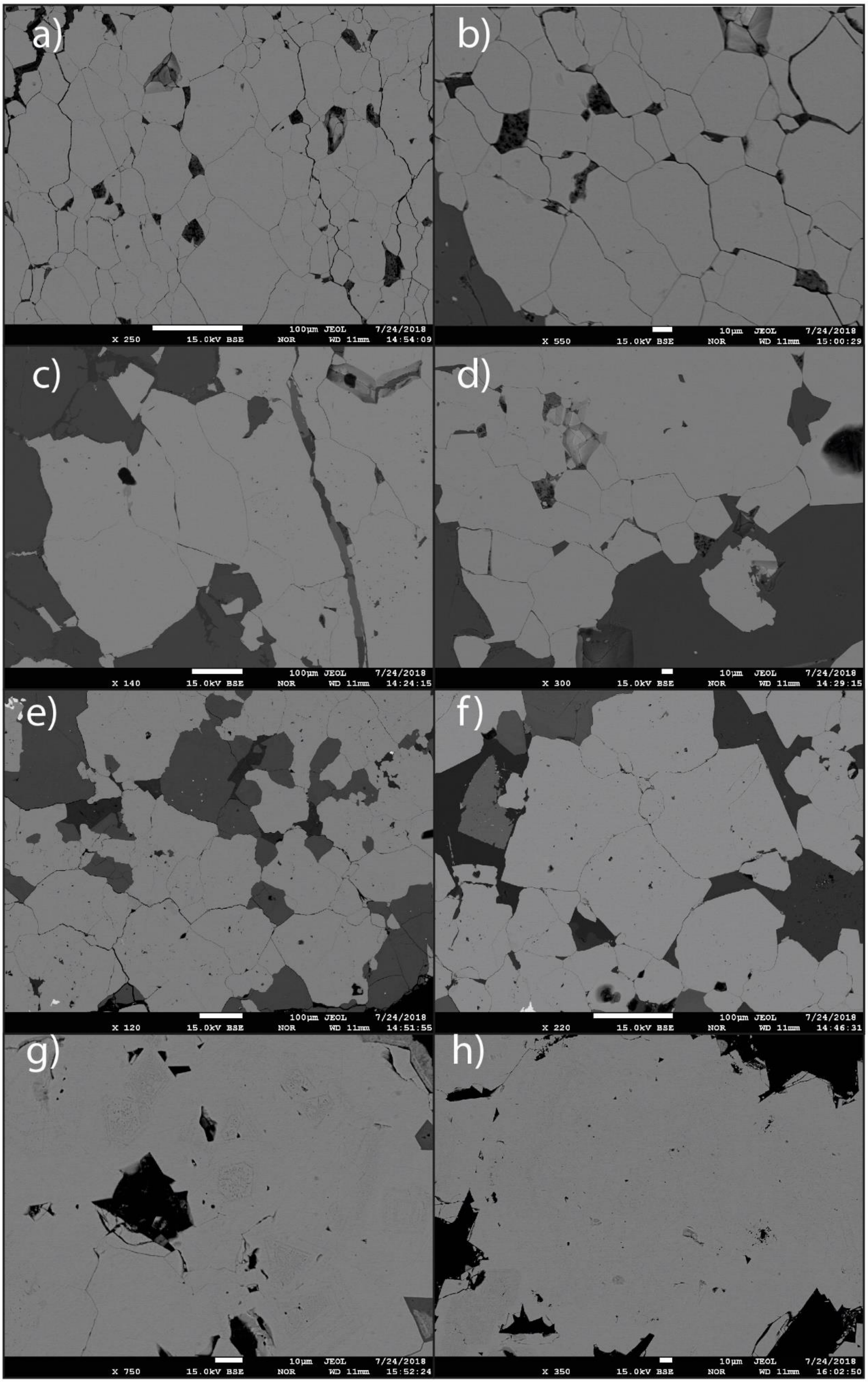

Supplementary Fig. 1
Greyscale BSE images of samples presented in Fig. 2, taken with a Field Emission-EPMA JXA-8530F JEOL hyperprobe. Except for some samples from El Laco, all chosen magnetite ores appear homogeneous with no discernable zonation or rims of alteration. Sample numbers: a) and b) K-MT-1079-303, c) and d) KES090020, e) 13-C-219 f), 13-C-219, g) and h) LS-11-4.

Supplementary Fig.2

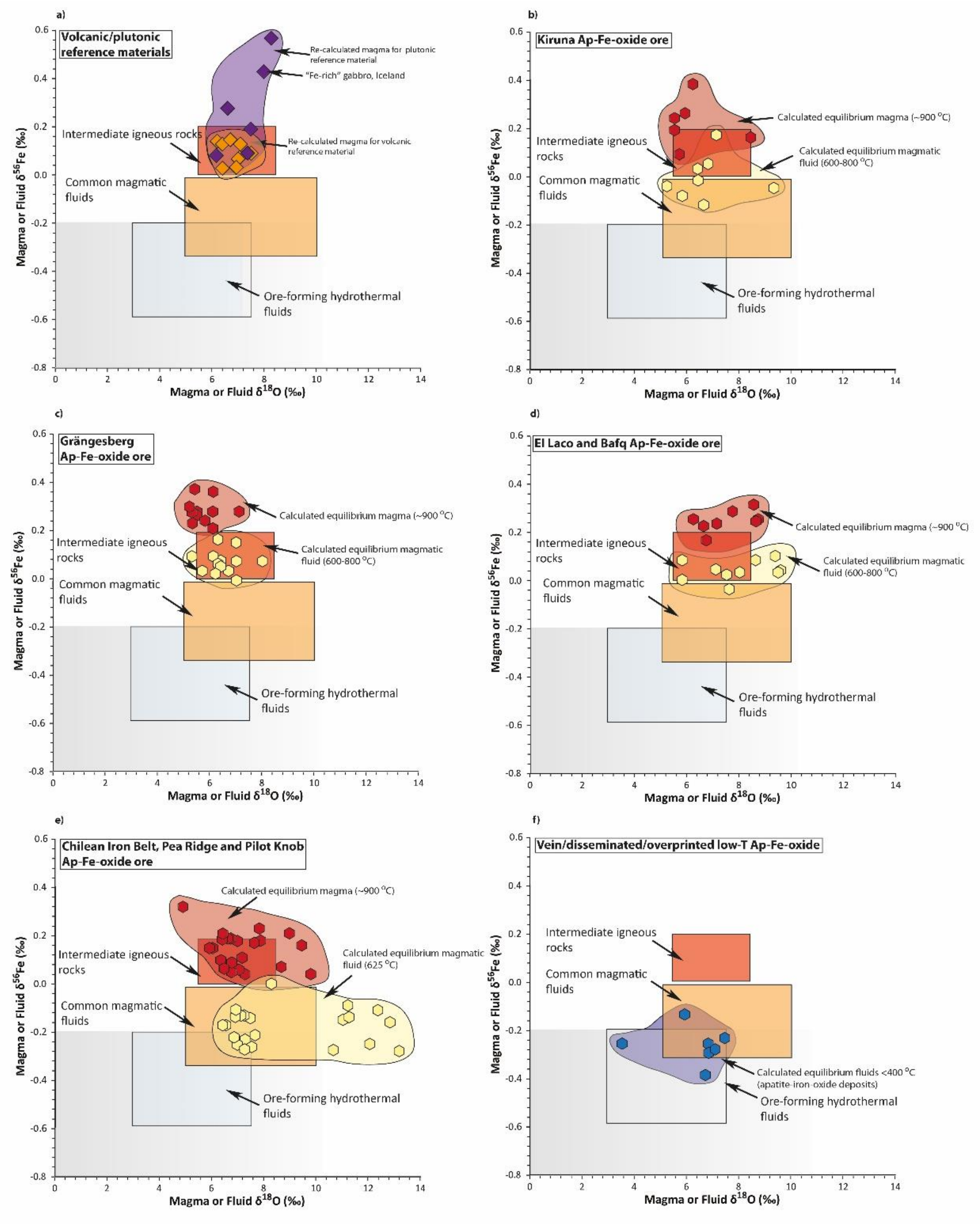

Supplementary Fig. 2

Equilibrium source calculations for Fe and O isotopes. a) Calculated isotopic compositions of magma in equilibrium with magnetite samples from volcanic and plutonic reference materials at magmatic T (> 800 C°). Calculated isotopic compositions of magma or fluid in equilibrium with magnetite samples from the Kiruna (b), Grängesberg (c), El Laco and Bafq Mining Districts (d) and Chilean Iron Belt, Pea Ridge and Pilot Knob apatite-iron oxide ore [14,48] compared to the reference fields for common magmatic and hydrothermal sources

[5,39,54,57,58,69,70,162,164–168]. Some of the calculated ore forming magmas and fluids that are in equilibrium with apatite-iron oxide ore magnetites are enriched in ^{56}Fe relative to common magmatic sources and plot above the currently accepted reference fields for intermediate magmas and high-temperature magmatic fluids. This may be the result of fractionation of the heavy isotope into the melt during early silicate crystallization in some mafic melts and consequently subsequent magnetite crystals may be enriched in the heavy iron isotope [46] (see text for details). Vein, disseminated and overprinted ore samples (Ve-Di) show equilibrium with common magmatic fluid sources only at low-temperatures (<400 °C), representing a secondary component under more hydrothermal conditions (f). For simplification only values with $\delta^{56}Fe \leq +0.6$ are plotted.
